# An integrated multiphase dynamic genome-scale model explains batch fermentations led by species of the *Saccharomyces* genus

**DOI:** 10.1128/msystems.01615-24

**Published:** 2025-01-22

**Authors:** Artai R. Moimenta, Diego Troitiño-Jordedo, David Henriques, Alba Contreras-Ruíz, Romain Minebois, Miguel Morard, Eladio Barrio, Amparo Querol, Eva Balsa-Canto

**Affiliations:** 1 Biosystems and Bioprocess Engineering, IIM-CSIC, Vigo, Spain; 2 Applied Mathematics II, University of Vigo 16784 , Vigo, Spain; 3 Applied Mathematics, University of Santiago de Compostela 16780 , Santiago de Compostela, Spain; 4 Yeastomics Laboratory, Food Biotechnology Department, IATA-CSIC, Paterna, Spain; University of Rhode Island, Kingston, Rhode Island, USA

**Keywords:** fermentation, yeast, dynamic flux balance analysis, time-varying cellular objective, secondary metabolism, *Saccharomyces *genus

## Abstract

During batch fermentation, a variety of compounds are synthesized, as microorganisms undergo distinct growth phases: lag, exponential, growth-no-growth transition, stationary, and decay. A detailed understanding of the metabolic pathways involved in these phases is crucial for optimizing the production of target compounds. Dynamic flux balance analysis (dFBA) offers insight into the dynamics of metabolic pathways. However, explaining secondary metabolism remains a challenge. A multiphase and multi-objective dFBA scheme (MPMO model) has been proposed for this purpose. However, its formulation is discontinuous, changing from phase to phase; its accuracy in predicting intracellular fluxes is hampered by the lack of a mechanistic link between phases; and its simulation requires considerable computational effort. To address these limitations, we combine a novel model with a genome-scale model to predict the distribution of intracellular fluxes throughout batch fermentation. This integrated multiphase continuous model (IMC) has a unique formulation over time, and it incorporates empirical regulatory descriptions to automatically identify phase transitions and incorporates the hypotheses that yeasts might vary their cellular objective over time to adapt to the changing environment. We validated the predictive capacity of the IMC model by comparing its predictions with intracellular metabolomics data for *Saccharomyces uvarum* during batch fermentation. The model aligns well with the data, confirming its predictive capabilities. Notably, the IMC model accurately predicts trehalose accumulation, which was enforced in the MPMO model. We further demonstrate the generalizability of the IMC model, explaining the dynamics of primary and secondary metabolism of three *Saccharomyces* species. The model provides biological insights consistent with the literature and metabolomics data, establishing it as a valuable tool for exploring the dynamics of novel fermentation processes.

**IMPORTANCE:**

This work presents an integrated multiphase continuous dynamic genome-scale model (IMC model) for batch fermentation, a crucial process widely used in industry to produce biofuels, enzymes, pharmaceuticals, and food products or ingredients. The IMC model integrates a continuous kinetic model with a genome-scale model to address the critical limitations of existing dynamic flux balance analysis schemes, such as the difficulty of explaining secondary metabolism, the lack of mechanistic links between growth phases, or the high computational demands. The model also introduces the hypothesis that cells adapt the FBA objective over time. The IMC improves the accuracy of intracellular flux predictions and simplifies the implementation process with a unique dFBA formulation over time. Its ability to predict both primary and secondary metabolism dynamics in different *Saccharomyces* species underscores its versatility and robustness. Furthermore, its alignment with empirical metabolomics data validates its predictive power, offering valuable insights into metabolic processes during batch fermentation. These advances pave the way for optimizing fermentation processes, potentially leading to more efficient production of target compounds and novel biotechnological applications.

## INTRODUCTION

Batch fermentation is widely used in industrial and food biotechnology to produce various products such as antibiotics, enzymes, and biofuels. It is also commonly used to make fermented foods and beverages such as wine, yogurt, bread, and beer (1). Batch fermentation occurs in several phases: lag, exponential growth, growth-no-growth transition, stationary, and decay. The lag phase is the period during which cells adapt to a new environment after inoculation. Yeast cells do not multiply, and their metabolic activity is low. Once adapted, cells enter the exponential growth phase, during which fermentation occurs at a constant, maximal rate. This phase is crucial for biomass growth and presents the maximum production rate of primary metabolism by-products, such as ethanol. The growth-no-growth (gng) transition occurs with nutrient starvation. Yeast cells switch from active growth to maintenance mode and sometimes exhibit secondary growth as a result of carbohydrate accumulation. Some secondary metabolites, including aromatic compounds, are produced during this phase, and their production continues during the stationary phase. Finally, in the death phase, the loss of viability is accompanied by a reduction in the fermentation rate. The duration of each phase depends on factors such as the microorganisms used, the specific medium, and environmental conditions (e.g., temperature, pH).

Genome-scale constrained-based models (GEMs) are commonly used to explore the phenotypic potential of the associated microorganism (2–4). GEMs impose environmental constraints using mass and energy balances, thermodynamics, and flux capacities. These constraints characterize all possible phenotypes of the microorganism. Flux balance analysis (FBA) can then determine the intracellular flux distribution corresponding to a particular biological objective such as maximum biomass growth or ATP production (5).

Dynamic implementations of flux balance analysis (dFBA) (6) have been successfully used to explain primary metabolism in batch fermentation. So far, most contributions reasonably explained the measured dynamics of biomass growth, carbon source uptake, and the production of relevant primary metabolites in alcoholic fermentation by *Saccharomyces cerevisiae* (7–10). However, the multiphase nature of the process and the fact that secondary metabolism is particularly relevant for generating flavors or aromas require alternative implementations that deal with these difficulties.

In a recent contribution, Henriques et al. (11) proposed a multiphase, multi-objective (MPMO) dynamic FBA implementation that successfully explained secondary metabolism and brought novel insights into how cold-tolerant yeast species achieve redox balance. The model has been applied to describe the phenotypic differences of various yeast species in batch fermentation (12, 13).

However, this modeling approach lacked a mechanistic connection between the phases, whose durations were otherwise estimated through data fitting. This limitation restricts the generality of the model. Additionally, the multiphase implementation is discontinuous, that is, it requires modifying the constraints and objective functions in every other phase, complicating the implementation and use of the model for non-experts.

To overcome these limitations, we propose an integrated multiphase continuous model (IMC model) combining a dynamic extracellular model that can automatically describe the different phases of batch fermentation with a dynamic genome-scale model to predict the distribution of intracellular fluxes. To do so, we extend the model proposed by Moimenta et al. (14) to predict secondary metabolism during batch fermentation, incorporating the role of transport of amino acids from the extracellular medium into the cell. The model includes a regulatory mechanism that can automatically detect fermentation phases. The resulting dynamic model can constrain an FBA implementation; hence, the biomass growth model also poses a constraint. At the same time, the cellular objective corresponds to a time-varying compromise between ATP and protein production.

The proposed IMC model was compared with the MPMO model in simulating fermentations led by *Saccharomyces uvarum,* and the results were validated using metabolomics data. Next, we applied the IMC model to explain the metabolism of three *Saccharomyces* species relevant for the food biotechnological industry (15): *S. cerevisiae* EC1118, *S. uvarum* BMV58, and *Saccharomyces kudriavzevii* CR85 in batch fermentation in a synthetic medium.

We validated the IMC model by comparing its predictions with intracellular metabolomics data for *S. uvarum* during batch fermentation. The model aligns well with the data, confirming its predictive capabilities. Notably, the IMC model predicts trehalose accumulation, a well-known mechanism in yeast fermentation, that needs to be enforced in the MPMO model. We further demonstrate the generalizability of the IMC model, explaining the dynamics of primary and secondary metabolism of three *Saccharomyces* species. We explored how these species metabolized carbon and nitrogen sources in the different phases. The model revealed differences in how cells produce energy and achieve redox balance and how these differences are translated into different exometabolome dynamics. Maximum differences appeared in the stationary phase, emphasizing the need for a model that is able to address phase transitions.

Overall, this model represents a significant advancement in the dynamic simulation of batch fermentation. Compared with the alternative MPMO model, it is easier to use, and it automatically accounts for all process phases and secondary metabolism and improves intracellular predictions while requiring fewer computational resources. It is a versatile tool that can be easily adapted to other species or strains and used to explore novel fermentations with alternative yeast species or engineer media or species for target products.

## RESULTS

### Implementation of the continuous multiphase dynamic genome-scale model

The IMC model relies on four essential components for its formulation: (i) time-series data of biomass and relevant external compounds, carbon and nitrogen sources, and, if possible, products; (ii) a dynamic kinetic model that describes the dynamics of the biomass and external metabolites to be used for constraining; (iii) the resolution of the dynamic FBA problem, and (iv) metabolic reconstruction of the microorganism under consideration.

#### Time-series data for model building

The nature of the data needed to build the model will largely depend on its objective. However, time-series data with approximately seven sampling intervals for biomass, substrates, and, potentially, products are required to calibrate a dynamic kinetic model. Furthermore, it is essential to ensure that the model is structurally identifiable (16).

In this work, we aim to elucidate the metabolic pathways underlying the phenotypes observed in various yeast species during batch fermentation. To this end, time-series data for biomass and exometabolome were used to formulate a dynamic kinetic model, whereas intracellular metabolomics data were used to validate the model results.

Data were obtained by sampling the bioreactors five to seven times to quantify biomass and exometabolites. Concerning biomass, we determined dry weight (gDW) and cell number (cells/L). Exometabolites correspond to sugars (glucose [Glx] and fructose [F]), amino acids available in the medium and metabolized by yeasts (alanine [Ala]; arginine [Arg]; aspartate [Asp]; cysteine [Cys]; glutamate [Glu]; glutamine [Gln]; glycine [Gly]; histidine [His]; isoleucine [Ile]; leucine [Leu]; lysine [Lys]; methionine [Meth]; phenylalanine [Phe]; serine [Ser]; threonine [Thr]; tyrosine [Tyr]; tryptophan [Try]; and valine [Val]), and ammonium (NH_4_Cl) and products obtained through fermentation including ethanol (Eth), glycerol (Gly), succinate (Succ), acetate (Ace), several secondary metabolites such as ethyl acetate (EthylA), isoamyl acetate (IamoA), phenyl ethyl acetate (PEA), isobutanol (Iobut), isoamyl alcohol (Iamo), 2-3 butanediol (BDO), 2-phenyl ethanol (PE), and malic acid (Mal) (all concentrations in g/L). Intracellular metabolomics data were collected at three sampling times in the exponential growth, gng, and stationary phases.

#### Formulation of the dynamic kinetic model

In this study, we formulate a model that builds on a previous one by Moimenta et al. (14). The model describes the role of amino acids and introduces several adaptations to account for experimental observations and automatically transition between the different fermentation phases. Detailed model equations can be found in Materials and Methods. Here, we describe the key mechanisms that are included in the final, most parsimonious model.

The biomass dynamics is explained by considering its composition in terms of carbohydrates, protein, and RNA, without decay or transport inhibition due to ethanol. After inoculation, the lag phase follows the Baranyi and Roberts model (17), affecting both biomass growth and uptake of assimilable nitrogen. During the fermentation process, yeasts assimilate nitrogen and sugars to generate biomass. The assimilable nitrogen, consisting of amino acids and ammonium, is the limiting substrate. A generalized mass action model represents the transport mechanism, whereas the growth rate adheres to the Monod model.

The transport of glucose and fructose, which are used for fermentation, is described using a Michaelis-Menten model. The proposed model integrates the progressive decline in the uptake rates of these sugars during the nitrogen starvation phase, which in agreement with previous research findings (18). Upon exhaustion of assimilable nitrogen sources, cells decelerate growth, leading to a transition to the stationary phase. During this phase, cells accumulate carbohydrates in the cytoplasm, consequently increasing cellular dry weight. This accumulation is incorporated as a secondary growth term in the biomass equation based on the model by Henriques and Balsa-Canto (19).

As cells enter the stationary phase as a result of nitrogen depletion, they undergo physiological, biochemical, and morphological modifications. The model incorporates an additional state to account for global regulatory mechanisms during this transition. During alcoholic fermentation, glucose and fructose are metabolized via the glycolytic pathway into several metabolites, including ethanol, succinate, acetate, glycerol, lactate, succinate, and 2-3 butanediol. The production of acetate esters (ethyl acetate, isoamyl acetate, and phenyl ethyl acetate) and higher alcohols (isobutanol, isoamyl alcohol, and 2-phenyl ethanol) has also been modeled. We proposed four distinct production models depending on the phase of compound release: proportional to sugar uptake (ethanol), gradually reduced over time following regulation (glycerol, acetate, and lactate), delayed until nitrogen depletion (succinate, ethyl acetate, isoamyl acetate, and isobutanol), or delayed until nitrogen depletion and slowly repressed during the stationary phase (ethyl acetate, phenyl acetate, 2-phenyl ethanol, and 2-3 butanediol).

Note that the model provides essential information to identify the different fermentation phases. The lag phase corresponds to the period in which growth does not reach a significant rate. Subsequently, the primary growth phase lasts until nitrogen sources are depleted, leading to secondary growth. The metabolic transition follows $\phi_{N,S}$ (equation 25). Finally, the stationary phase is reached when the cell growth rate turns numerically null.

#### Formulation of the dynamic flux balance analysis

The formulation of the dynamic flux balance analysis problem uses a genome-scale yeast model. Specifically, we used the extended version of the yeast8 model (20), as proposed in reference 11, which incorporates previously absent metabolites and reactions to describe secondary metabolism.

We used the continuous model (equations 9–26) to constrain the exchange reactions of the genome-scale model as follows:


(1) $$v_{L}=v_{U}=v_{X}=\frac{\frac{dX}{dt}}{{MW}_{X}}\cdot 1,000$$

where $v_{X}$ represents the flux of a certain compound X (i.e., carbon sources, nitrogen sources, and all products included in the dynamic model) expressed in mmol/(DW ·h^−1^). ${MW}_{X}$ regards the metabolite molecular weight. However, we allowed for an inequality in the lower bound for compounds whose measurements present higher relative experimental noise. This is the case with histidine, succinate, lactate, malate, isobutanol, isoamyl alcohol, 2-phenyl acetate, and 2-phenylethanol.

The biomass flux constraint reads as follows: equations 5–8 as


(2) $$v_{L}=v_{U}=v_{X}=\frac{\frac{dX}{dt}}{X}$$

We modeled the maintenance of ATP associated with growth (GAM), taking into account the energetic polymerization costs of the different macromolecules, that is, proteins, nucleic acids, and carbohydrates, as:


(3) $${GAM=GAM}_{b}+GAM_{Prot}+GAM_{RNA}+GAM_{Carbs}$$

where GAM*_b_* is a modifiable parameter that quantifies ATP costs in growth for non-polymerization cellular processes. In our case, we adopted GAM*_b_* = 30 mmol · gDW^−1^ in agreement with the previous literature (21).

We used a parsimonious implementation of flux balance analysis with several cellular objectives: maximizing biomass (biomass not being a constraint in this case), maximizing ATP, maximizing a combination of ATP and protein, maximizing a combination of accumulation of ATP, and carbohydrates. In all four cases, we computed the flux scores of biomass normalized by the phase duration and compared the results with the expected values, computed using the kinetic model fitted to the experimental data. None of these formulations succeeded: maximization of biomass resulted in no growth during the exponential phase, maximizing ATP resulted in lack of growth, maximizing the combination of ATP and protein, underestimates growth in the exponential phase, and overestimating growth in the gng phase while maximizing a combination of ATP and carbohydrate accumulation underestimates growth throughout the process (fluxes are reported in Table S1).

We then hypothesized that cells could vary the objective over time to represent a microbial adaptation to the changing environment under batch conditions. To represent this adaptation, we proposed that the objective is for yeast to initially maximize ATP production, which is essential for growth and maintenance. Once nitrogen becomes limiting, cells can no longer produce new proteins at the same rate and start to recycle the existing proteinogen pool, which is then used preferentially for synthesizing enzymes involved in stress response and survival. This time-varying objective is mathematically formulated as follows:


(4) $$J\left(t\right)=\max\left(J_{ATP}\cdot (1-\phi_{N,S})+J_{P}\cdot \phi_{N,S}\right)$$

Since regulatory mechanisms, gene expression, and enzyme activity change over time, metabolic fluxes adjust to meet evolving cellular demands. The optimal flux distribution corresponds to that maximizing J(t) and minimizing the overall flux as in pFBA.

### The continuous model offers advantages over the discontinuous model

**Fig 1:**
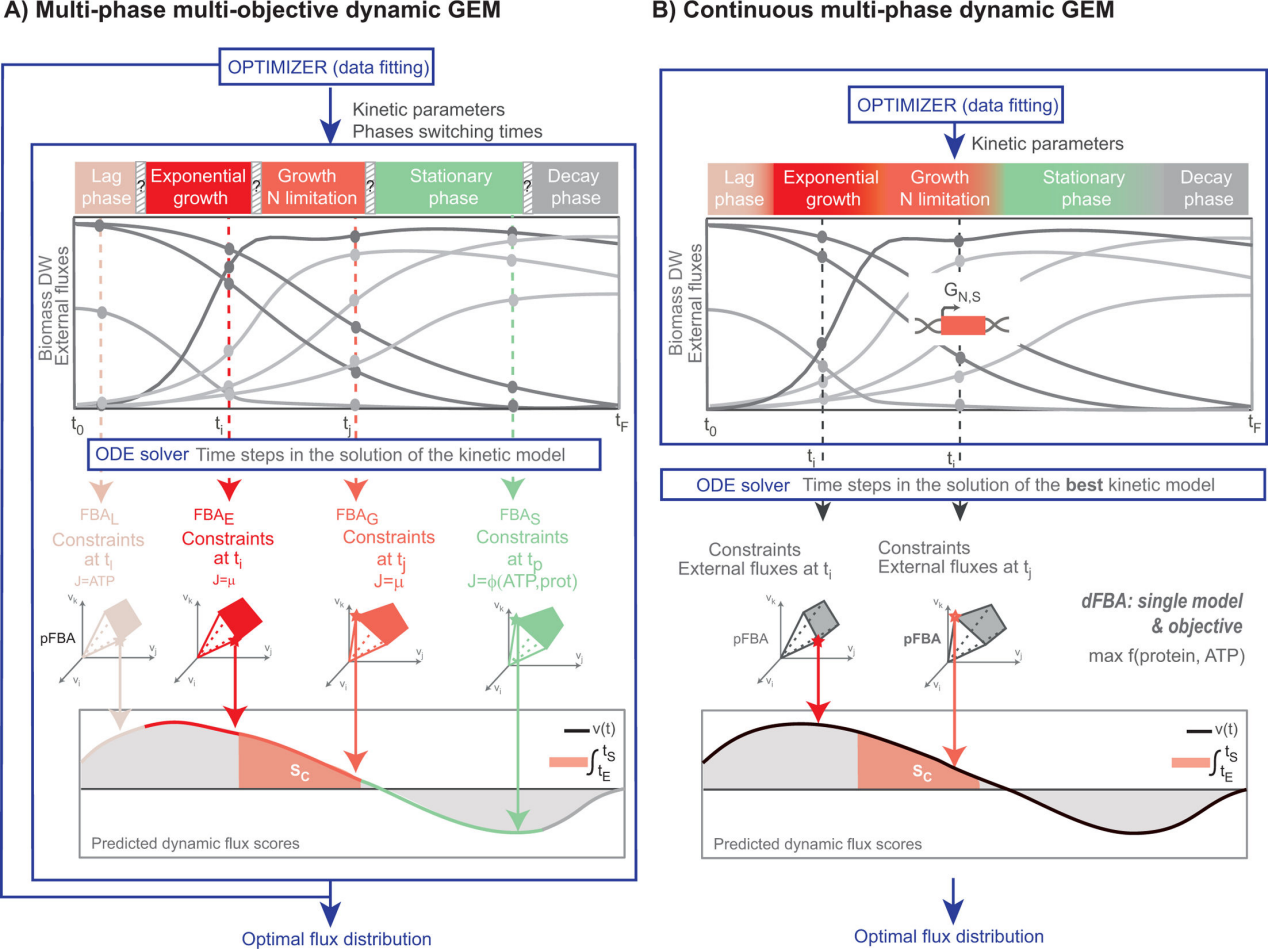
multiphase multi-objective vs continuous model solution. (**A**) In the MPMO model, data fitting and dFBA are coupled. The FBA problem is solved at each time step used by the ordinary differential equation solver (ODE solver) every time the least squares function is evaluated within the data fitting. The FBA problem formulation changes from phase to phase. (**B**) In the IMC model, the dFBA is solved using the best kinetic model. The FBA problem is solved at each time step used by the ordinary differential equation solver (ODE solver) only once the model has been fitted. A unique FBA problem formulation is solved over time, although the cost function is dynamic.

As shown in Fig. 1, although the components to build the MPMO model are mostly the same, the IMC and MPMO models differ in two crucial aspects. First, they differ in the calculation of transition times between phases; second, they differ in the formulation of the FBA problem. In the MPMO model, the transition times between phases are considered model parameters, estimated together with kinetic parameters by data fitting. Moreover, the constraints and the objective function of the dFBA problem vary with the phases. The parameter estimation problem and dFBA are resolved simultaneously. This implies that to solve the data fitting problem (a non-linear dynamic optimization problem), the FBA problem (a constrained linear optimization problem) is solved internally at each discretization time used by the ordinary differential equation solver. Conversely, the IMC model uncouples data fitting from the FBA problem; the model that best fits the data automatically determines transition times, which serve as external constraints for the dFBA problem over time. Consequently, it is not only simpler to implement and solve but also more computationally efficient.

### The continuous model shows good predictive capabilities

To validate the IMC model, we first compared its quality to recover the dynamics of *S. uvarum* BMV58 (SU-BMV58) in batch fermentation at 25°C. This allowed for a direct comparison with the MPMO model proposed by Henriques et al. (11). In that work, the fermentation medium included sucrose. Therefore, we incorporated its dynamics into the IMC model. Additionally, the equations describing the dynamics of acetate and succinate account for the consumption observed in the data set. The IMC model consists of 41 ordinary differential equations that depend on 46 unknown parameters. The model was fitted using time series data for biomass, nitrogen sources, glucose, fructose, sucrose, and products such as ethanol, glycerol, acetate, succinate, higher alcohols, and acetate esters. The mean values of three replicates were used for data fitting. Note that we considered the experimental error of each data as computed from the experimental replicates. Therefore, the model tends to fit better the data with a lower standard deviation.

The continuous model successfully captured the process dynamics for all observables, except those with a low signal-to-noise ratio in the HPLC data (namely, 1-hexanol, lysine, and cysteine), for which the discontinuous model also failed. The optimal parameter values and the *R*-squared goodness of fit for all observables can be found in Data Set S1.1. The mean, median, and variance of the *R*-squared values of both models are similar, with a slightly better mean value for the continuous model (*R*^2^ = 0.93 versus *R*^2^ = 0.91). To further compare both models, we performed the Kolmogorov-Smirnov test using the corresponding *R*-squared values of both models to contrast their statistical distributions. The results did not reveal statistical evidence that both R-squared value distributions differed at any reasonable confidence level (*P*-value ≈ 0.72). Therefore, it can be concluded that both models are equivalent in goodness-of-fit.

To compute internal metabolic fluxes, we used the metabolic reconstruction in Henriques et al. (11) and implemented the dFBA with the IMC model. After solving the dFBA problem, flux scores (FS*_r_*) and normalized flux scores (NFS*_r_*) for each reaction *r* were computed using time integrals and normalized time integrals for each phase, following equation 36. Figure S1 in the supplemental material shows normalized flux scores during the growth and stationary phases for central carbon metabolism, illustrating how metabolic activity is reduced over time.

Furthermore, we compared the predictions of the IMC model with the metabolomics data collected at three different time points during fermentation (data are presented in Data Set S1.3). Although metabolomics data are relative, we used them to identify the presence or absence of metabolites. The IMC model can recover the presence of all metabolites in all phases. Interestingly, the IMC model activates the trehalose-6-phosphate (T6P) pathway (reactions r_0195 and r_1051 in Data Set S1.4) that leads to trehalose accumulation found in metabolomics data (Data Set S1.3). In contrast, the MPMO model does not activate this pathway unless a constraint is imposed, and even in this case, the model does not predict the observed trehalose accumulation in the stationary phase.

### The IMC has good generalization capabilities

To address the generalizability of the model, we tested its performance in describing fermentations led by three species at 20°C: *S. cerevisiae* EC1118, *S. uvarum* BMV58, and *S. kudriavzevii* CR85, regarded as SC-EC1118, SU-BMV58, and SK-CR85 from now on.

**Fig 2:**
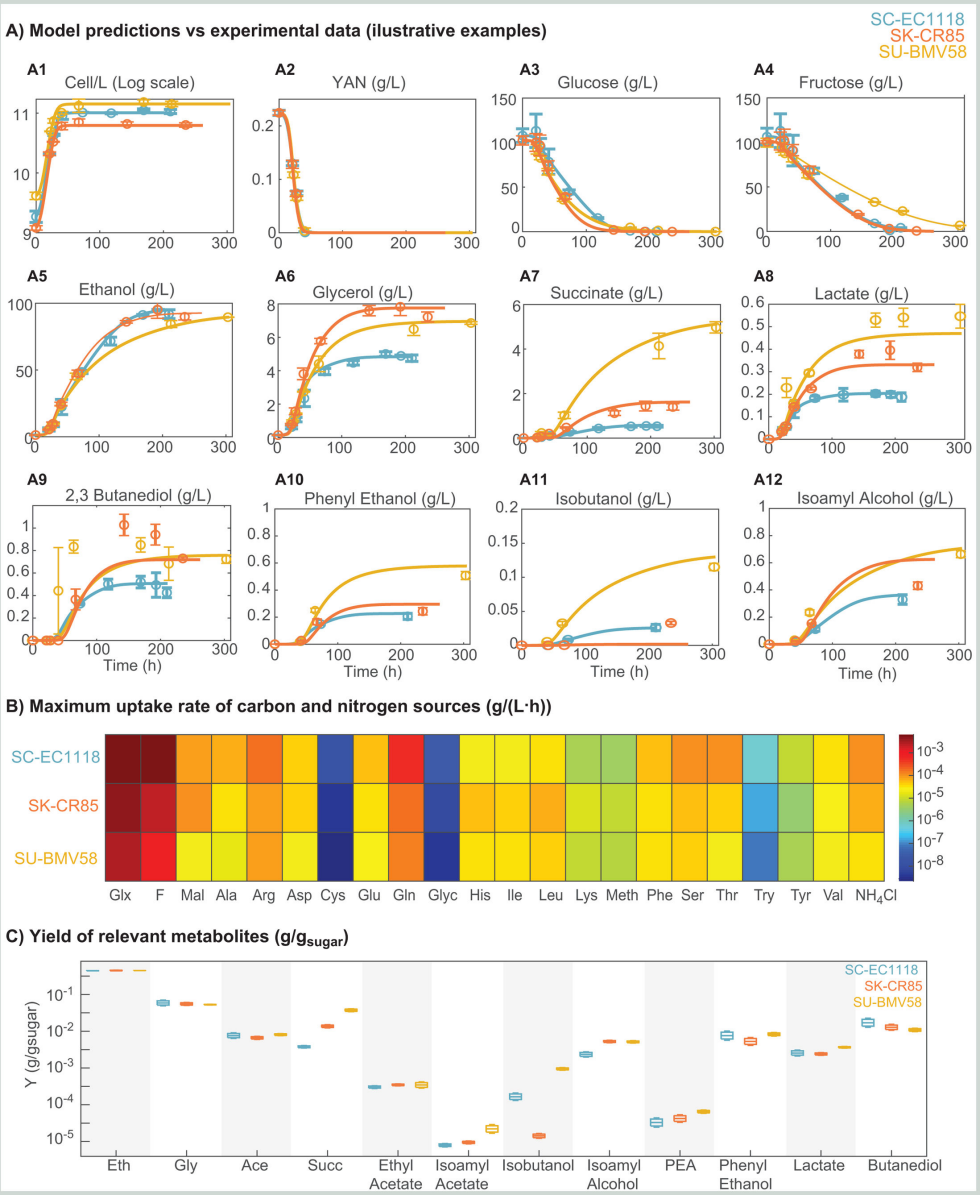
Continuous model performance. (**A**) Illustrative examples of the IMC model fit to the data. Continuous lines show model predictions. Circle symbols indicate experimental data points, and error bars are based on standard deviation as obtained from experimental replicates. (**B**) Maximum uptake rate of carbon and nitrogen sources in g/(L⋅h) showing the different preferences for the different species. (**C**) Yields obtained for relevant metabolic products with their associated uncertainties. Substantial differences are observed in the yields of succinate, isoamyl acetate, isoamyl alcohol, phenylethyl acetate (PEA), or isobutanol for the three species.

We first fitted the dynamic kinetic model to time series data as described above. We evaluated the *R*-squared value of goodness of fit for each species and observable, and the mean and median were above *R*^2^ = 0.936 and *R*^2^ = 0.98, respectively, for all species. All values are reported in Data Set S1.2. The experimental data versus the corresponding model predictions for some relevant observables are presented in Fig. 2A1–12; the optimal parameters and the model versus time series data for all observables can be found in the supplemental material.

We then used the dynamic kinetic model to constrain the dynamic flux balance analysis framework to decipher the most parsimonious intracellular metabolic fluxes and pathways behind these extracellular phenotypes. The consensus genome-scale reconstruction Yeast8 of *S. cerevisiae* S288C (v.8.7.0) (20) was used as a basis to generate metabolic reconstructions of the species. The reconstruction was extended in previous work to incorporate reactions related to the production of secondary metabolites (11). The metabolic reconstruction of SK-CR85 was obtained by comparing its genome sequence with the reference strain *S. cerevisiae* S288c (13). SC-EC1118 was sequenced, *de novo* assembled, and finally annotated with YGAP (22). We compared its genome with the reference *S. cerevisiae* S288C. We performed a presence/absence of genes analysis based on the functional annotation provided by YGAP. In summary, a reference strain gene is considered present in SC-EC1118 if YGAP gave it a systematic name. If this was not the case, this was considered a “new” gene (present in EC1118 but not in S288C). The new genes were functionally annotated with blastKoala (23). As no loss of functional genes was observed in the genome of the EC1118 strain, compared with what is considered in the reference model (see details in Data Set 2.1), we used the consensus metabolic reconstruction yeast8. Simulated dynamic flux scores (FS) are reported in Data Sets S2.2–4 as mmol of the metabolite produced per mmol of consumed hexose × 100.

### Different species show different batch fermentation dynamics

Figure 2A presents the dynamics of cell number (cell/L), uptake, and production of several compounds for the three species. Figure 2A1 shows that SU-BMV58 achieves the maximum number of cells at the end of the process, whereas SK-CR85 achieves the minimum. This is the first indication that different species use resources and tolerate batch fermentation stressors differently.

The IMC model revealed that the species differ substantially in phase durations. The duration of the exponential growth phase for SU-BMV58 is twice the duration of SC-EC118 (21, 31, and 42 h for SC-EC1118, SK-CR85, and SU-BMV58, respectively). The gng phase spans 49, 31, and 57 h for SC-EC1118, SK-CR85, and SU-BMV58, respectively. The duration of the stationary phase is 130 h for SC-EC1118, 192 h for SK-CR85, and 155 h for SU-BMV58.

Differences in phase duration are mainly related to differences in substrate uptake rates (Fig. 2A2–A4). SC-EC1118 is the quickest in uptaking nitrogen sources, whereas SU-BMV58 is the slowest. The preferences in nitrogen sources are similar between species (Fig. 2B). Cysteine and glycine are the least preferred of all nitrogen sources with uptake rates between $2.2\times 10^{-9}g/(L\cdot h)$ and $1.6\times 10^{-8}g/(L\cdot h)$ for all species. Glutamine and arginine are the most preferential, with uptake rates between $8.3\times 10^{-5}g/(L\cdot h)$ and $5.6\times 10^{-4}g/(L\cdot h)$. The dynamics of individual amino acids are shown in Fig. S6.

The IMC model also shows considerable differences in the case of carbon source uptake rates (Fig. 2A3, A4, and B). SC-EC1118 appears to have the highest uptake rates for both glucose and fructose (uptake rates: $6.4\times 10^{-3}g/(L\cdot h)$ and $5.6\times 10^{-3}g/(L\cdot h)$, respectively) than the other species, glucose being the preferred carbon source for these three species. In contrast, SU-BMV58 has the lowest hexoses uptake rates ($2.9\times 10^{-3}g/(L\cdot h)$ and $9.6\times 10^{-4}g/(L\cdot h)$ for glucose and fructose, respectively). Note that fermentation with SU-BMV58 is significantly longer due mainly to the slower uptake of fructose (see Fig. 2A3 and A4).

The amounts of products and the production dynamics are also different between species. Figure 2A5–A12 and C present the dynamics and yields of the relevant metabolic products. All species produce substantial amounts of ethanol (>88 g/L; Fig. 2A5), but SC-EC1118 produces slightly more in a shorter fermentation. In contrast, SK-CR85 and SU-BMV58 produce higher amounts of glycerol, a temperature stress protector. SK-CR85 produced up to 7.5 g/L, SU-BMV58 around 7 g/L, whereas SC-EC1118 barely produced 5 g/L (see Fig. 2A6). SU-BMV58 showed up to 3.6 times the succinate yield compared with SC-EC1118 (see Fig. 2A7 and C). All species show low lactate production (between 0.2 g/L produced by SC-EC1118 and 0.5 g/L for SU-BMV58; see Fig. 2A8). In addition, cold-tolerant species show enhanced production of higher alcohols (see Fig. 2A9–A12). SK-CR85 and SU-BMV58 produce similar amounts of 2,3 butanediol (around 0.8 g/L) and isoamyl alcohol (around 0.6 g/L), whereas SC-EC1118 produced 0.8 g/L of butanediol, and around 0.3 g/L of isoamyl alcohol. SU-BMV58 produced almost twice the amount of phenyl ethanol than the other species that produce similar amounts; SU-BMV58 is also the highest producer of isobutanol, barely produced by SC-EC1118 and SK-CR85.

#### The dynamics of central carbon metabolism differs between cold-tolerant species and *S. cerevisiae*

**Fig 3:**
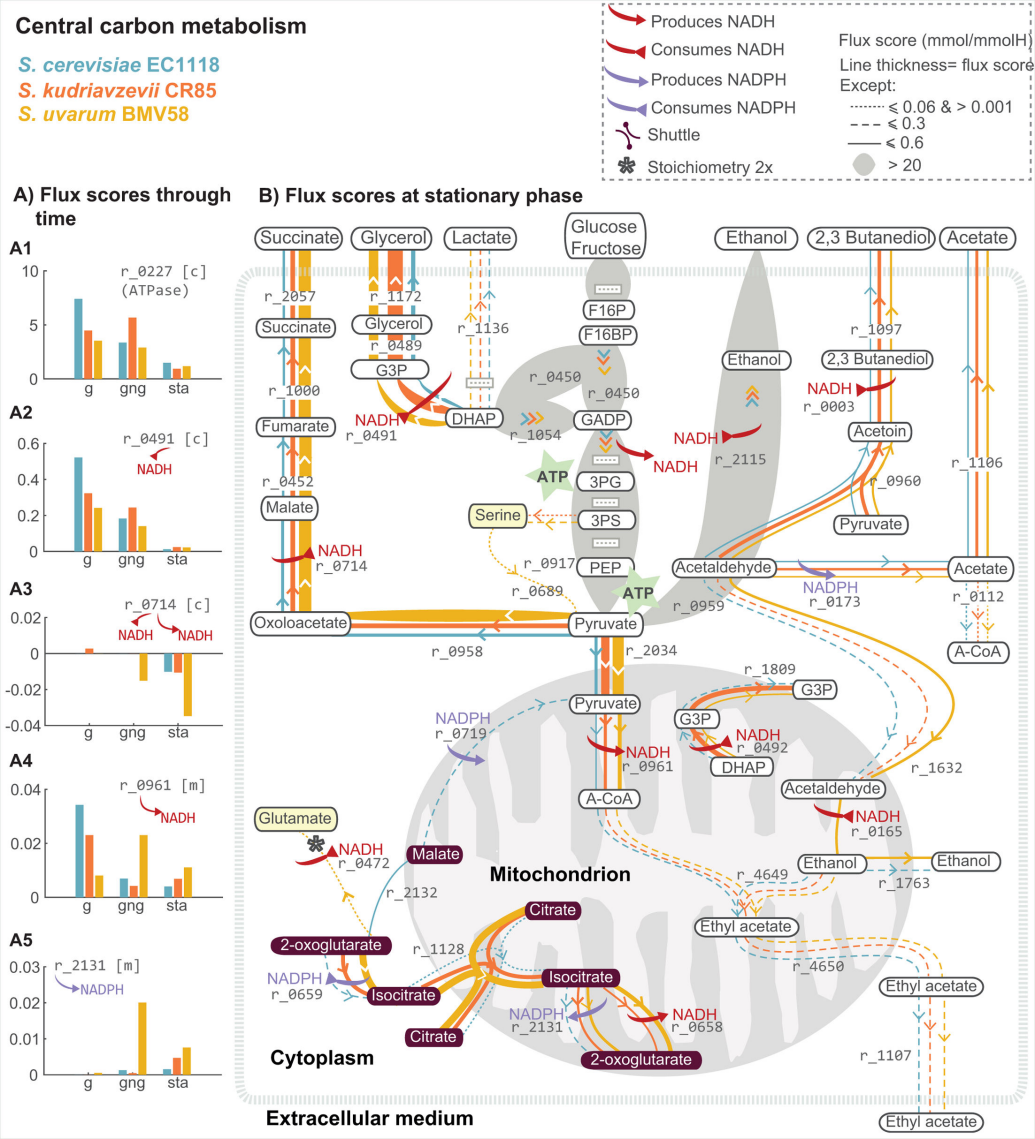
Comparison of predicted flux scores in ATP production and redox balance for selected species. Panel A presents the flux scores calculated for growth (g), growth-no growth (gng), and stationary (sta). These flux scores are computed following equation 36 from the start to the end of each phase, normalized by the phase duration. The duration of the exponential growth phase is 21, 31, and 42 h for SC-EC1118, SK-CR85, and SU-BMV58, respectively. The transition between growth and non-growth phases lasts 49, 31, and 57 h, and for SC-EC1118, SK-CR85, and SU-BMV58. The stationary phase lasts 130 h for SC-EC1118, 192 h for SK-CR85, and 155 h for SU-BMV58. Panel B illustrates the predicted intracellular dynamic flux scores (≥0.01 mmol/mmol H) during the stationary phase.

**Fig 4:**
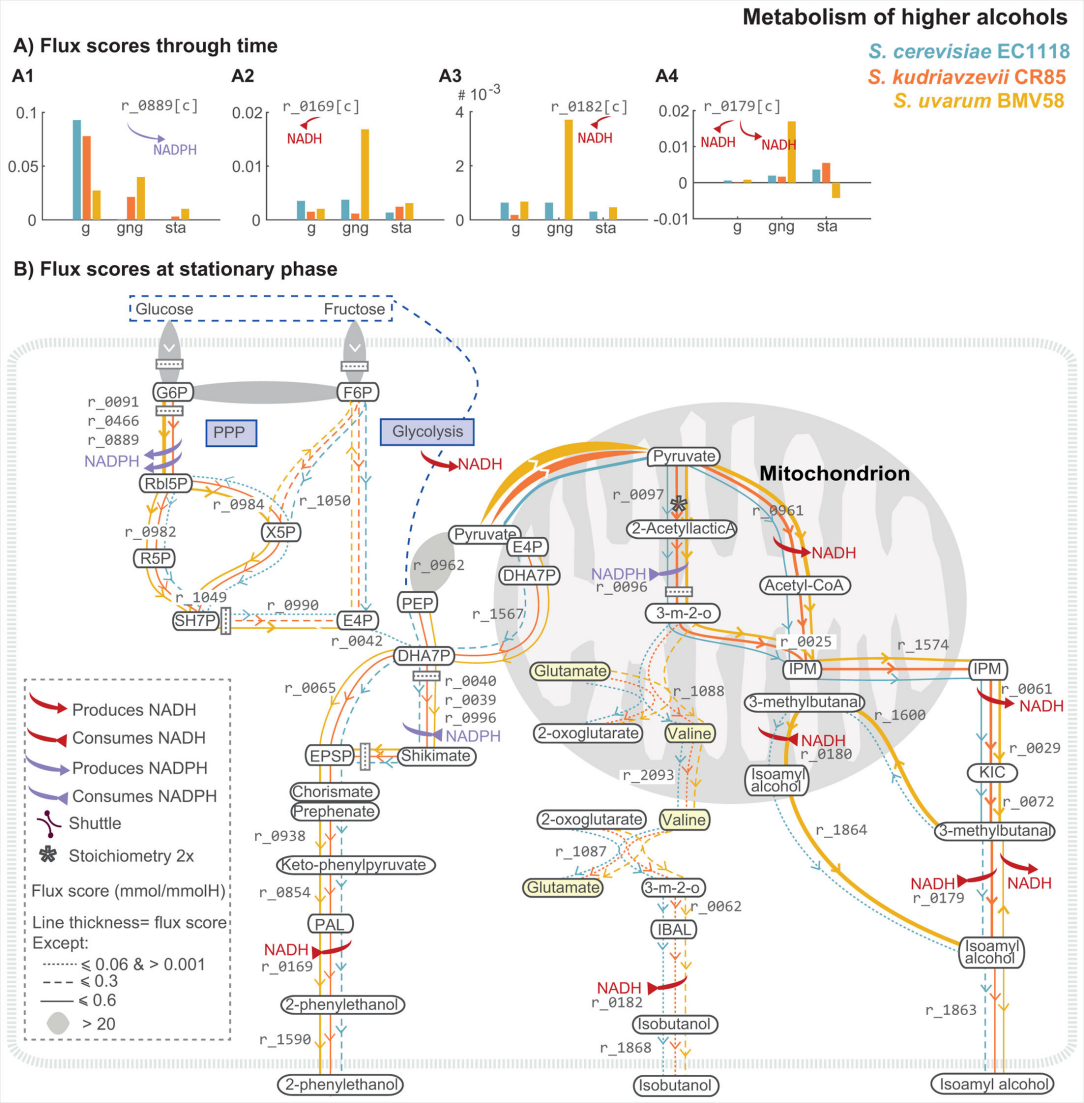
Comparison of predicted flux scores corresponding to PPP and higher alcohol production by selected species. (≥0.01 mmol/mmol H) related to the production of higher alcohols 2–phenyl ethanol, isobutanol, and isoamyl alcohol during the stationary phase as well as the role of different pathways leading to the redox balance for the different species.

The model revealed significant differences between species in their use of metabolic pathways related to energy production and redox balance. Figures 3A1–A5 and 4A1–A4 show flux scores in different fermentation phases normalized by phase duration. Since the most significant differences among species were observed during the stationary phase, Fig. 3B and 4B show the flux scores for the reactions satisfying $\left|\log_{10}\left(\left|S_{1}/S_{2}\right|\right)\right|\geq 10^{-3}$, where $S_{1}$ and $S_{2}$ correspond to the flux score of a reaction for species 1 and 2. All flux scores can be found in Data Set 2.5.

In anaerobic conditions, yeast cells rely mainly on glycolysis to metabolize hexoses. This process involves breaking down hexoses into pyruvate, generating ATP (r_0892 and r_0962) and NADH in the process (r_0486). Because dissolved oxygen is consumed by cells very rapidly, pyruvate is converted mainly to ethanol through alcoholic fermentation (sequence of reactions r_0959 and r_2155). This conversion regenerates NAD+, which is essential for the continuation of glycolysis. However, the three species also use two alternative routes for pyruvate. A portion is carboxylated to form oxaloacetate (r_0958) whereas another portion is transported to the mitochondria (r_2034). We explore these routes in more detail in the sequel.

ATP production is practically the same for all species, with total scores ranging between 186.37 for SK-CR85 and 186.91 for SU-BMV58. The enzyme ATPase catalyzes the hydrolysis of ATP, releasing energy, which the cell can use for various functions. The energy production flux scores related to this hydrolysis of ATP (Fig. 3A1, r_0227) vary with time differently for the three species. Although the trend is similar, the maximum score appears in the growth phase, but for SK-CR85. Afterward, the scores show a gradual decline. However, some differences among the strains are noted; in the case of SK-CR85, the highest score is achieved in the gng transition. Interestingly, SK-CR85 presents the shortest gng transition and the highest rate of uptake of hexoses in this phase ($1.8182\frac{mmol}{mmolH\cdot h}$ for SU-BMV58, $2.0408\frac{mmol}{mmolH\cdot h}$ for SC-EC1118, and $3.2258\frac{mmol}{mmolH\cdot h}$ for SK-CR85).

The redox couple NADH/NAD^+^ is at the interface between anabolism and catabolism, ultimately coupling together reactions which are, in most cases, parts of different pathways, and sometimes even taking place in different organelles. For instance, during glycolysis, glycerol-3-phosphate oxidation is coupled to the reduction of NAD^+^ to NADH (r_0486). The required NAD^+^${}^{+}$is produced using different metabolic pathways, depending on the species and the fermentation phase. As evidence of different management of these cofactors between the three strains, notable divergences appear in reactions r_0491, r_0714, r_0961, and r_2131 (see Fig. 3A2–A4).

In r_0491 (Fig. 3A2), the NADPH-dependent enzyme, glycerol-3-phosphate dehydrogenase, catalyzes the reduction of DHAP to G3P. The flux score for reaction r_0491 is markedly higher for SC-EC1118 during the growth phase, whereas SK-CR85 has the highest flux score during the gng and stationary phases. This observation in SK-CR85 is related to the substantial production of glycerol by cold-tolerant species, which allows the reoxidation of excess NADH (produced by glycolysis) to NAD^+^. A similar observation is reported in r_0492 in mitochondria during the stationary phase (see Fig. 3B).

As mentioned before, all three species divert a portion of the produced pyruvate to form oxaloacetate (r_0958). This is particularly relevant for SU-BMV58. The model predicts an increase in flux score diverted to this reaction over time, achieving the maximum flux score of 5.30 mmol/mmol H during the stationary phase. SK-CR85 and SC-EC1118 also use this pathway but to a lesser extent (maximum scores achieved at the stationary phase are 1.32 and 2.03 mmol/mmol H, respectively). The subsequent reaction r_0714 (Fig. 3A3) produces or consumes NADH, depending on the phase and species involved. All species reduced oxaloacetate to malate during the stationary phase using NADH as a reducing agent (r_0714:1.32,2.03,5.38). SC-EC1118 shows a considerable flux score exclusively during the stationary phase, which correlates to late succinate production. SU-BMV58 shows the highest flux scores during gng and stationary phases, which explains its markedly higher succinate production (see Fig. 2A5).

A third portion of pyruvate is transported to mitochondria. Maximum flux scores through this reaction are observed in the stationary phase. Differences between phases are particularly notable for SU-BMV58, presenting a net FS of 1.85 mmol/mmol H during the exponential growth, 3.8 mmol/mmol H during the gng, and 4.9 mmol/mmol H during the stationary. A fraction of pyruvate in the mitochondria is converted to acetyl-CoA with the release of NADH r_0961 (see Fig. 3A3 and B). This NADH is then used for different purposes in the mitochondria, such as the production of G3P (mostly in SK-CR85) or ethanol (SU-BMV58) as well as the production of higher alcohols or their precursors (Fig. 4).

After the NADH/NAD^+^ pair, NADPH/NADP^+^ is the second pair of nicotinamide-based cofactors, crucial in cellular anabolism reactions, principally. As illustrated in Fig. 4, the primary source of NADPH during fermentation is the pentose phosphate pathway. Nevertheless, the IMC model indicates that the three species also generate NADPH in the mitochondria through the conversion of isocitrate to 2-oxoglutarate r_2131. Flux scores for SU-BMV58 are notably higher than those for SC-EC118, which barely uses this pathway, and SK-CR85, which mainly uses this pathway during the stationary phase. The NADPH produced in the mitochondria is used to reduce keto acids to dihydroxy acids in the synthesis of branched-chain amino acids (r_0096, Fig. 4B).

#### Cold-tolerant species use the pentose phosphate pathway (PPP) and mitochondrial pyruvate to achieve redox balance enhancing the production of aromatic compounds

Figure 4A1 and B shows significant differences in the use of the pentose phosphate pathway by cold-tolerant species. SC-EC1118 uses this pathway mostly during exponential growth, whereas SK-CR85 and SU-BMV58 use PPP throughout fermentation (see Fig. 4A1). In stationary phase, SU-BMV58 shows the highest flux scores (r_0466,r_0889:0,0.60,1.59; Fig. 4A1 and B). The PPP provides ribose-5-phosphate for nucleotide synthesis and erythrose-4-phosphate (E4P, r_0990:0.059,0.35,0.69) for the synthesis of aromatic amino acids. Note that only SC-EC1118 uses the reaction r_0042 to synthesize 7-phospho-2-dehydro-3-deoxy-D-arabino-heptonic acid (DHA7P) in the cytoplasm, whereas the preferred route is to transport E4P to mitochondria, synthesize DHA7P, which is then transported to the cytoplasm (r_0020,r_1567:0.12,0.47,0.48 in stationary phase) acting as a precursor of shikimate and chorismate synthesis. Subsequently, through several steps, phenylacetaldehyde (PAL) is produced and transformed into 2-phenyl ethanol using NADH (r_1590:0.18,0.47,0.48, in stationary phase). 2-Phenyl ethanol is finally released into the medium, with SU-BMV58 being the maximum producer (Fig. 2A10).

A fraction of pyruvate in the mitochondria is used to produce 2-acetyl lactic acid, which is then transformed into (R)-2,3-dihydroxy-3-methylbutanoate (3-m-2-o) using NADPH (r_0096). The dynamics of this reaction vary with species: SC-EC1118 shows the maximum FS among species during exponential growth [0.006 mmol/(mmolH⋅H)]. In contrast, SU-BMV58 shows the maximum FS between species during the stationary phase [0.009 mmol/(mmolH⋅H)]. A fraction of 3-m-3-o is then used to produce valine *de novo*, and once in the cytosol, it is transformed into isobutanol through the Elrich pathway using NADH (see Fig. 4B). Figure 4A3 shows the dynamics of the flux scores corresponding to the last step in this transformation. Interestingly, the scores for SK-CR85 are low, indicating a low isobutanol production, whereas the scores for SU-BMV58 are exceptionally high during the gng. These flux scores would explain the substantial increase in the isobutanol production rate for SU-BMV58 late in fermentation observed in Fig. 2A11.

Another fraction of mitochondrial pyruvate is transformed into acetyl-CoA, which is partially used to produce isoamyl alcohol through 2-isopropylmalate (IPM; r_0025, Fig. 4B). Subsequent steps lead to the production of 3-methylbutanal, which is used differently by the three different species. SK-CR85 uses NADH to reduce 3-methylbutanal to isoamyl alcohol mainly in the gng and stationary phases with NFS 0.0016 and 0.0055 mmol/(mmolH⋅H), respectively (r_0179, Fig. 4A4 and B). In SC-EC1118, 3-methylbutanal follows two different paths, the preferential route, the reduction to isoamyl alcohol in the cytoplasm r_0179, with a maximum NFS 0.0036 mmol/(mmolH⋅H) in the stationary phase, and the transport and reduction to isoamyl alcohol in mitochondria r_0180 with a maximum NFS $7.7\times 10^{-5}\;mmol/(mmolH\cdot H)$. SU-BMV58 uses a different strategy in the stationary phase (Fig. 4A4 and B). 3-Methylbutanal is transported to mitochondria where it is reduced to isoamyl alcohol, consuming NADH at the step. The isoamyl alcohol is then transported to the cytoplasm and partially to the extracellular medium (r_1863) and partially used to produce 3-methylbutanal while producing NADH (r_0179). Note that the highest net flux to the extracellular medium corresponds to SU-BMV58 in all phases (r_1863:0.03,0.93,1.05 mmol/(mmolH⋅H) during growth, gng and stationary phase, respectively).

## DISCUSSION

In this study, we proposed an integrated multiphase continuous genome-scale model (IMC) capable of detecting and explaining the different phases in yeast batch fermentation. Our model is based on an adaptation of the dynamic kinetic model proposed by Moimenta et al. (14), which is then used to constrain a multiphase implementation of dFBA.

Henriques et al. (11) proposed the first dynamic genome-scale model to explain secondary metabolism in yeast fermentation. The underlying idea was to use a multiphase multiobjective formulation of the problem (MPMO), whose formulation changes from phase to phase. Unlike the MPMO model, the IMC offers a single formulation over time to predict intracellular flux distributions across all fermentation phases without manually defining phase transitions. In addition, the IMC uncouples the solution of the kinetic model parameter estimation and the dFBA problems, thus substantially improving computational efficiency. The IMC also avoids the need for separate phase definitions and phase-specific objectives and constraints, making it easier to implement than the MPMO model.

The resolution of the dFBA problem requires the definition of an appropriate cellular objective function. Notably, the selection of such a function remains an open question. Static single-, multi-objective, and lexicographic approximations have been proposed to elucidate primary metabolism (24, 25). In the present study, static functions were insufficient to elucidate secondary metabolism. The proposed time-varying objective function represents the biological balance between energy generation and protein turnover once nitrogen is exhausted. This adaptability is crucial in dynamic systems such as batch fermentation, where cells undergo significant changes in metabolism over time in response to the depletion of nutrients and the accumulation of metabolites. Although our approach is heuristic, it provides a practical and biologically relevant means to model phase-dependent metabolism, complementing existing dFBA methods and offering a new perspective on dynamic metabolic behavior.

To solve the dFBA problem, we chose a direct approach. Although the direct approach is still widely used, it is well acknowledged that it is subject to certain limitations, such as the potential for flux solutions to exhibit discontinuities. Alternatives, such as those proposed by Gomez et al. (25), Scott et al. (26), or de Oliveira et al. (27) would prevent discontinuous solutions. However, these methodologies presuppose that cells optimize a static objective function or a concatenated series of static objectives, rendering these methods unsuitable for the IMC model. Although our intracellular flux estimation does not explicitly enforce continuity between consecutive time steps, it should be noted that the use of pFBA, the large number of external constraints, and the smooth evolution of the objective function mitigate the appearance of discontinuities. Note that, as shown in the flux variability analysis (Data Set 2.10), the use of pFBA significantly reduces the variability in the solution space, in all phases, thus contributing to more consistent and biologically plausible flux predictions. Alternative numerical approaches for the IMC model are being explored. However, in dynamic systems, some degree of flux variability is expected and may reflect genuine shifts in metabolic priorities, particularly considering that aromatic compounds are produced late in the process.

We have analyzed the reliability of the IMC model, comparing model results with intracellular metabolomics data in batch fermentations led by *S. uvarum* BMV58 (28). The IMC model can predict metabolism effectively, as evidenced by its ability to predict trehalose accumulation, whereas the MPMO model requires this production to be enforced by internal constraints. Remarkably, trehalose metabolism is crucial for stress tolerance and growth regulation (29). The ability to recover trehalose production showcases the robustness of the IMC model in capturing secondary metabolism processes.

We have also shown that the model is generalizable by exploring the metabolism of three different yeast species, *S. cerevisiae* EC1118, *S. uvarum* BMV58, and *S. kudriavzevii* CR85, in batch fermentation, under the same conditions (synthetic medium and 20°C). The model was able to recover the extracellular data with high accuracy. The IMC model demonstrated differential uptake rates of carbon and nitrogen sources, which have implications for the metabolic efficiency of each species. In alignment with the literature, the estimated glucose uptake rates surpassed those of fructose for the three species, with the *S. cerevisiae* strain showing a faster consumption of glucose compared to cold-tolerant species (30–33). With respect to nitrogen substrates, the highest uptake rates observed were for glutamine and arginine across the three strains. This finding aligns with the positions of these two amino acids as the main sources and donors of nitrogen in grape juice and corroborates the general classification of amino acids based on their quality to support yeast growth (34–36).

Similarly, the model showed clear differences in the yields of glycerol, succinate, or isoamyl alcohol, also widely documented in the literature (28, 37–39). These differences can be routed to different metabolic strategies that the different species used to achieve redox balance, particularly during the gng and stationary phases, which is consistent with our previous observations (11, 13, 28).

However, in contrast to our previous works, the model did not identify the GABA shunt as the main metabolic pathway involved in the increase in succinate production during the stationary phase. Instead, most succinate would be produced through the TCA reductive branch in all phases, although the flux scores vary with time and species. In addition to yielding succinate as an end product, the GABA shunt produces the NADPH cofactor. Henriques et al. (11, 13) suggested that the increase in the flux through the GABA shunt is due to the need for NADPH for lipid biosynthesis through the consumption of acetate and mevalonate production from the beginning of the stationary phase. However, we did not observe acetate uptake here. One possible explanation is the fermentation medium: natural grape juice vs. synthetic medium used in the present work. It is plausible that the natural grape juice used by Henriques et al. (11, 13) had a lower or limiting lipid content compared with our synthetic must (1.5 mg/L beta-sitosterol; Girardi-Piva et al. [40]). Under our non-limiting lipid conditions, the biosynthesis of lipids from acetate and mevalonate, as suggested by Henriques et al. (11), is more energy-intensive than the direct uptake of exogenous intermediates. Consequently, the flux through the GABA shunt becomes secondary or unnecessary. This hypothesis coincides with the low expression of glutamate decarboxylase encoded by GAD1—the first step in the GABA shunt—observed by Bach et al. (41) in synthetic grape must with non-limiting anaerobic factors (7.5 mg/L ergosterol, 2.5 mg/L oleic acid, and 0.21 g/L TWEEN80). Note also that the objective function of the IMC includes the maximization of ATP production, which is undoubtedly more efficient in the cytoplasm; therefore, the IMC model would divert lower flux to mitochondria, reducing flux through the mitochondrial GABA shunt. NADPH would then be obtained through the pentose phosphate pathway. Another possible explanation is that phase durations are slightly different when estimated by fitting data in the MPMO model and using the IMC model. When estimated as parameters in the MPMO model, their values had an associated uncertainty of up to 20%. Therefore, the use of the GABA shunt in the different phases of fermentation requires further analysis.

The model also showed a significantly higher flux toward mitochondria in the case of *S. uvarum* and a higher production of higher alcohols during the gng and stationary phases for both *S. kudriavzevii* and *S. uvarum* compared with *S. cerevisiae*. Again, these results agree with previous findings (11, 13, 28). Note that higher alcohol production contributes to achieving the cellular redox balance for these species. Importantly, the estimated fluxes of pyruvate to the synthesis of aromatic compounds in mitochondria support the observation by Crépin et al. (42) that higher alcohols and their esters are from newly synthesized precursors rather than from the catabolism of exogenous amino acids.

The fact that internal fluxes are consistent with previous modeling and experimental results leads us to conclude that the proposed continuous dFBA approach allows us to link yeast genotypes with context-specific phenotypes under batch fermentation conditions. The model automatically accounts for phase transitions and uses a single formulation of constraints and objective functions within the FBA approach, facilitating its use. We showed that it is essential to address the different phases as metabolism varies substantially with time. This is particularly important in explaining the production of primary metabolites such as glycerol or succinate, whose production dynamics vary between *S. cerevisiae* and cold-tolerant species, and also in the prediction of the production of aromatic compounds in the gng and stationary phases.

Considering all phases of the process, our model provides an accurate explanation of primary and secondary metabolism and an improved understanding of the underlying mechanisms of batch fermentation. The proposed modeling concept is versatile and can be applied to other yeast species or microorganisms relevant to food and pharmacological biotechnology.

## MATERIALS AND METHODS

### Experimental materials and methods

#### Yeast strains

In this study, three yeast strains were utilized, specifically strains belonging to the species *S. cerevisiae*, *S. uvarum,* and *S. kudriavzevii*. The three strains used were *S. cerevisiae* EC1118 (denoted as SC-EC118), *S. uvarum* BMV58 (denoted as SU-BMV58), and *S. kudriavzevii* CR85 (denoted as SK-CR85). SC-EC1118 is a commercial wine strain that was originally isolated from wine production in France. SU-BMV58 is another commercial wine strain, whereas SK-CR85 is a natural isolate obtained from an oak tree’s bark. The strains were cryogenically preserved at −80°C and cultured and maintained on GPY plates (2% glucose, 2% agar, 0.5% peptone, and 0.5% yeast extract). GPY plates were kept at 4°C to preserve the strains.

Prior to inoculation, an overnight starter culture was prepared by pouring a small amount of biomass from the plate into an Erlenmeyer flask with 25 mL of GPY liquid medium (2% glucose, 0.5% peptone, and 0.5% yeast extract) and placed at 25°C with agitation under aerobic conditions.

### Data for IMC model validation

For the comparison of the MPMO and IMC models, we used the data set previously used to formulate the MPMO model corresponding to *S. uvarum* BMV58 in batch fermentation at 25°C Henriques et al. (11). In the present work, we have also used intracellular metabolomics data to validate model predictions. Data correspond to the relative abundance extracellular metabolites collected at three sampling times, one per phase, exponential, growth-no-growth transition, and stationary. A detailed description of how intracellular metabolites were extracted and quantified can be found in Minebois et al. (28).

### Data for IMC model generalization

#### Fermentations and sampling

Detailed information on the experimental design and the sampling methods can be found in Contreras-Ruiz et al. (43). Time series data were obtained from fermentations carried out in temperature-controlled bioreactors at 20°C. The bioreactors were filled with 470 mL of synthetic must, following the same recipe as in Rollero et al. (44), and inoculated at OD-600 0.1 (approximately $1\cdot 10^{6}$ cells / mL) of the starter cultures. For each strain, three independent biological replicates were performed.

Samples were collected across the fermentation, covering the different phases of fermentation. At each sampling point, dry weight (DW), residual hexoses, residual amino acids and ammonium, organic acids, the main fermentative by-products (e.g., ethanol, succinate, glycerol), and volatile compounds were quantified. Cell density (C/mL) was measured using a MUSE cell analyzer (Millipore). Dry weight biomass was obtained by measuring the weight difference of a pre-weighted filter (0.45 µm pore size) used to filtrate 5 mL of broth, rinsed twice with 50 mL of deionized water and placed for 48 h at 110°C. Determination of amino acids and ammonia (yeast assimilable nitrogen) was carried out following the same protocol as the one followed by Su et al. (45). Sugars (glucose and fructose), fermentative by-products (glycerol, ethanol, 2-3 butanediol, and erythritol), and organic acids (acetate, succinate, tartrate, citrate, and malate) were determined using an HPLC (Thermo Fisher Scientific, Waltham, MA) equipped with a refraction index and UV/VIS (210 nm) detector. Samples were diluted and filtered through a 0.22-µm nylon filter (Symta, Madrid, Spain). Metabolite separation was performed through a HyperREZTM XP Carbohydrate H+ 8 mm column fitted with a HyperREZTM XP Carbohydrate Guard (Thermo Fisher Scientific, Waltham, MA). The mobile phase consisted of 1.5 mM of H2SO4 at a flux of 0.6 mL/min, and the HPLC oven was kept at 50°C.

### Theoretical methods

#### Dynamic continuous model

The model builds upon a previously developed model by Moimenta et al. (14). The model accounts for the following state variables: biomass (X) ($g_{DW}/L$); protein content in biomass ($X_{P}$) ($g_{P}/L$); carbohydrate content in biomass ($X_{C}$) ($g_{C}/L$); mRNA content in biomass ($X_{mRNA}$) ($g_{mRNA}/L$); glucose and fructose (Glx and F); ethanol (Eth; g/L); glycerol (Gly); succinate (Succ); acetate (Ace); several secondary metabolites such as ethyl acetate (EthylA), isoamyl acetate (IamoA), phenyl ethyl acetate (PEA), isobutanol (Iobut), isoamyl alcohol (Iamo), 2-3 butanediol (BDO), 2-phenyl ethanol (PE), and malic acid (Mal). All the metabolites are measured in g/L. The model accounts for assimilable nitrogen (YAN) ($g_{N}/L$) including ammonia (NH_4_Cl) ($g_{NH4Cl}/L$), and the different amino acids measured in g/L present in the medium: alanine (Ala), arginine (Arg), aspartate (Asp), cysteine (Cys), glutamate (Glu), glutamine (Gln), glycine (Gly), histidine (His), isoleucine (Ile), leucine (Leu), lysine (Lys), methionine (Meth), phenylalanine (Phe), serine (Ser), threonine (Thr), tyrosine (Tyr), tryptophan (Try), and valine (Val). Note that yeasts do not metabolize proline under anaerobic conditions; therefore, we do not include it as an assimilable nitrogen source.

The model was built following an iterative procedure. Here we present the original, most complete model, which accounts for cellular decay:

- Differential equations:
  Biomass production. Biomass is modeled taking into account its major components: carbohydrates, protein, and mRNA. Their dynamics are described as follows:
(5) $$\frac{dX_{C}}{dt}=\lambda_{C}\cdot \mu_{N}+\mu_{C}-k_{D}\cdot X_{C}\cdot \phi_{ED}$$
(6) $$\frac{dX_{P}}{dt}=\lambda_{P}\cdot \mu_{N}-k_{D}\cdot X_{P}\cdot \phi_{ED}$$
(7) $$\frac{dX_{mRNA}}{dt}=(1-\lambda_{C}-\lambda_{P})\cdot \mu_{N}-k_{D}\cdot X_{mRNA}\cdot \phi_{ED}$$
    where $\lambda_{C}=0.29$, $\lambda_{P}=0.59$, and $1-\lambda_{C}-\lambda_{P}=0.12$ correspond to the percentage of carbohydrate, protein, and mRNA in biomass, respectively. We used the values reported by Schulze et al. (18) for nitrogen-limited batch fermentation (59% protein and 12% mRNA); $\mu_{N}$ and $\mu_{C}$ (1/h) regard primary and secondary growth; and $k_{D}\cdot \phi_{ED}$ represent the cellular decay due to the production of ethanol.
    The biomass equation results:
(8) $$\frac{dX}{dt}=\frac{dX_{C}}{dt}+\frac{dX_{P}}{dt}+\frac{dX_{mRNA}}{dt}$$
  Nitrogen uptake.
(9) $$\frac{dYAN}{dt}=-\frac{\mu_{N}}{Y_{x/N}}$$
    $Y_{xN}(g_{DW}/g_{N})$ is the yield of nitrogen to biomass.
  Ammonia and amino acids uptake follows a generalized mass action model:
(10) $$\frac{d[{NH}_{4}Cl]}{dt}=-lag\cdot k_{{NH}_{4}Cl}\cdot [{NH}_{4}Cl]\cdot X_{P}$$
    and
(11) $$\frac{d[{AA}_{i}]}{dt}=-lag\cdot k_{{AA}_{i}}\cdot [AA_{i}]\cdot X_{P}$$
    where we denoted by $\left[{AA}_{i}\right]$ the extracellular concentration of each amino acid in g/L and $k_{{AA}_{i}}$ ($1/(g_{prot}\cdot h)$) is the associated kinetic parameter.
  Sugars uptake. The medium contains both glucose and fructose. Sucrose can be added, and it breaks down into glucose and fructose. The uptake is described following the Michaelis-Menten model. Note that the uptake rate varies when nitrogen is still available in the medium.
(12) $$\frac{dSucrose}{dt}=-k_{Sucrose}\cdot Sucrose$$
(13) $$\frac{dGlx}{dt}=-v_{max,Glx}\cdot X_{P}\cdot \frac{Glx}{Glx+ks_{Glx}}\cdot (1+\alpha \cdot \mu_{N})-\frac{1}{2}\frac{dSucrose}{dt}$$
(14) $$\frac{dF}{dt}=-v_{max,F}\cdot X_{P}\cdot \frac{F}{F+ks_{F}}\cdot (1+\alpha \cdot \mu_{N})-\frac{1}{2}\frac{dSucrose}{dt}$$
    Where $v_{max,Glx}$ and $v_{max,F}$ correspond to the maximum uptake rates for glucose and fructose, respectively, in g/L. $ks_{Glx}$ and $ks_{F}$ correspond to the Michaelis-Menten constants. The term $\alpha \cdot \mu_{N}$ describes the increase in the production of sugar transporters during primary growth, which results in a higher uptake rate of sugars during that phase. $k_{Sucrose}$ ($h^{-1}$) correspond to the rate degradation sucrose parameter.
  Gene regulation. An empirical expression describes the transition to the stationary phase once YAN and sugars are being depleted:
(15) $$\frac{dG_{N,S}}{dt}=(\phi_{N,S}\cdot \phi_{Sugar}-G_{N,S})\cdot \tau_{G_{N,S}}$$
    $\phi_{N,S}$ and $\phi_{Sugar}$ are smooth sigmoidal functions used to activate secondary growth and transition to the stationary phase (see their definitions later in this section). $\tau_{G_{N,S}}$ controls the velocity of those transcriptional changes.
  Extracellular primary metabolites production.
    The rate of excretion of metabolites is directly proportional to the glucose and fructose uptake. The yield coefficient ($Y_{i}$, where i represents Eth, Gly, Ace, Succ) expressed in ($g_{i}/g_{sugar}$) varies significantly depending on the fermentation phase. We use smooth logistic type functions to describe this variation, depending on whether there is an increase in production associated with the transition to secondary growth ($Y_{i}\cdot \phi_{N,S}$) or a decrease in production associated with the transition to the stationary phase ($Y_{i}\cdot (1-G_{N,S})$). δ is a binary parameter that allows us to accommodate the dynamics of acetate depending on whether the yeasts show a phenotype of consumption associated with nitrogen starvation in natural or synthetic must. It takes a value of 1 for synthetic must and 0 for natural must.
(16) $$\frac{dEth}{dt}=-Y_{Eth}\cdot \left(\frac{dGlx}{dt}+\frac{dF}{dt}\right)$$
(17) $$\frac{dGly}{dt}=-Y_{Gly}\cdot \left(\frac{dGlx}{dt}+\frac{dF}{dt}\right)\cdot (1-G_{N,S})$$
(18) $$\begin{array}{rl} \frac{dAce}{dt}= & -\delta \cdot Y_{Ace}\cdot \left(\frac{dGlx}{dt}+\frac{dF}{dt}\right)\cdot (1-G_{N,S})\\ & +(1-\delta )\cdot [-Y_{Ace}\cdot \left(\frac{dGlx}{dt}+\frac{dF}{dt}\right)\cdot (1-\phi_{N,S})\\ & -kc_{Ace}\cdot \phi_{N,S}\cdot X_{P}\cdot Ace\cdot (1-G_{N,S})] \end{array}$$
(19) $$\frac{dSucc}{dt}=-Y_{Succ}\cdot \phi_{N,S}\cdot \left(\frac{dGlx}{dt}+\frac{dF}{dt}\right)-kc_{Succ}\cdot (1-\phi_{N,S})\cdot Succ\cdot X_{P}$$
  Extracellular secondary metabolites production.
    Analogous expressions are used to describe secondary metabolites. Metabolites modeled were acetic esters (ethyl acetate, isoamyl acetate, and phenyl ethyl acetate), higher alcohols (isobutanol, isoamyl alcohol, and 2-phenyl ethanol), and 2,3 butanediol.
(20) $$\frac{dP_{i}}{dt}=\left\{ \begin{array}{l} -Y_{i}\cdot \phi_{N,S}\cdot \left(\frac{dGlx}{dt}+\frac{dF}{dt}\right)\\ or\\ -Y_{i}\cdot \phi_{N,S}\cdot \left(\frac{dGlx}{dt}+\frac{dF}{dt}\right)\cdot (1-G_{N,S}) \end{array}\right.$$
    The best candidate in equation 20 was selected for each product through model calibration.
  Experimental data revealed that under tested conditions, cells uptake malic acid, which we described following a generalized mass action model:
(21) $$\frac{dMal}{dt}=-kc_{Mal}\cdot Mal\cdot X_{P}$$
    where $k_{c,Mal}$ ($1/(g_{prot}\cdot h)$) is the associated kinetic parameter.
- Constitutive equations:
  Lag phase.
(22) $$lag(t)=\frac{a_{0}}{a_{0}+(1-a_{0})\cdot e^{-\mu_{MaxN}\cdot t}}$$
    Analytical solution of the commonly accepted model by Baranyi and Roberts (17). $a_{0}$ is a parameter bounded between 0 and 1 and represents the physiological state of the inoculum. µ_MaxN_ is the maximum specific growth rate ($h^{-1}$). This function enables the transition from the lag phase to the exponential growth phase.
  Primary growth.
(23) $$\mu_{N}=v_{AA}\cdot Y_{x/N}=14.0067\cdot \sum_{i=1}^{n}\frac{{NN}_{i}}{{MW}_{i}}\frac{d[{AA}_{i}]}{dt}\cdot Y_{x/N}$$
    $\mu_{N}$ describes the specific growth rate associated with cell division. We defined growth considering the proportion of nitrogen present in the amino acids and ammonia. ${NN}_{i}$ and ${MW}_{i}$ refer to the number of nitrogen atoms and molecular weight present in the molecule of each nitrogen compound (amino acid and ammonia), respectively, for example, arginine: $NN_{Arg}=4$; $MW_{Arg}=174.2g/mol$.
  Secondary growth.
(24) $$\mu_{C}=\phi_{N,S}\cdot X\cdot \Phi_{C}\cdot (\Theta_{C}-\frac{X_{C}}{X})$$
    $\mu_{C}$ describes the secondary growth using a mechanism for carbohydrate accumulation that was modeled with an empirical expression analogous to a proportional controller (19). $\Theta_{C}$ represents a set point for the ratio of carbohydrates in the biomass content ($\frac{X_{C}}{X}$), and $\Phi_{C}$ controls the velocity of the convergence towards that point. The activation of the secondary growth was modeled using the function ($\phi_{N,S}$):
(25) $$\phi_{N,S}=1-\frac{YAN}{YAN+k_{sC}}$$
    $\phi_{N,S}$ regulates the nitrogen concentration needed to induce carbohydrate accumulation and the transcriptional changes associated with nitrogen starvation. $k_{sC}$ is the half-saturation constant in g/L. Note that $\phi_{N,S}$ corresponds to a sigmoidal function bounded between [0,1]. Its value is close to 0 at the beginning of the process while nitrogen sources are still available and rapidly becomes 1 fully activating the secondary growth.
  Transition to the stationary phase.
    Cells transition to the stationary phase once nitrogen has been depleted and sugars are gradually being uptaken. We defined a function $\phi_{Sugar}$, which regulates the sugar concentration needed to induce the transcriptional changes associated with the stationary phase.
(26) $$\phi_{Sugar}=1-\frac{Glx+F}{Glx+F+k_{sS}}$$

#### Model calibration

The model in equations 5-26 depends on more than 40 unknown parameters, which are to be estimated from experimental data. The so-called model calibration problem aims to find the values of the unknown parameters that minimize (or maximize) some distance between model predictions and experimental data, subject to the system dynamics and parameter bounds (46). Hence, the optimal parameters correspond to those that maximize the (log-)likelihood function:


(27) $$J=\log(\Pi (\hat{y}|\theta ))$$

Under the assumptions of independently identically distributed measurements according to a Gaussian law for a given sampling time $t_{i}$, the maximization of J is equivalent to minimizing:


(28) $$J_{llk}(\theta )=\sum_{i=1}^{n_{exp}}\sum_{d=1}^{n_{d}}\frac{(y^{d}-y^{m}{)}^{2}}{\sigma_{d}^{2}}$$

where $n_{exp}$ corresponds to the number of experiments; $n_{d}$ corresponds to the number of data per experiment; $y^{m}$ and $y^{d}$ regard the model predictions and the measured data, respectively, and $\sigma_{d}$ is the standard deviation associated with the experimental data as computed from the experimental replicates.

The confidence interval ($\rho_{i}$) associated with each parameter estimate may be obtained through the covariance matrix:


(29) $$\pm t_{\alpha /2}^{\gamma }\sqrt{C_{ii}}$$

where $t_{\alpha /2}^{\gamma }$ is given by Student’s *t*-distribution, $\gamma =N_{d}-\eta$ degrees of freedom, and (1−α)100% is the confidence interval selected, typically 5%.

For non-linear models, there is no exact way to obtain C. Therefore, approximations have been suggested. Possibly the most widely used is based on the Crammèr-Rao inequality, which establishes, under certain assumptions on the number of data and non-linear character of the model, that the covariance matrix may be approximated by the inverse of the Fisher information matrix (F) in its typical definition:


(30) $$F=E\left([\frac{\partial J_{llk}(\theta )}{\partial \theta }{]}^{T}[\frac{\partial J_{llk}(\theta )}{\partial \theta }]\right)$$

#### Parsimonious flux balance analysis

Metabolic fluxes in the cell are obtained using a constraint-based model derived from flux balance analysis (FBA) (5, 47). FBA is a modeling framework based on the metabolic network stoichiometry and a steady-state mass balance condition:


(31) S⋅v=0

where S is the stoichiometric matrix of (*n* metabolites by *m* reactions) and v is a vector of metabolic fluxes. The number of unknown fluxes is higher than the number of equations, and thus, the system is undetermined. Still, it is possible to find a unique solution under the assumption that cell metabolism complies with the maximization (or minimization) of a certain cellular objective (J):


(32) max J

(33) s.t. :

(34) S⋅v=0

(35) $$v_{lb}<v<v_{ub}$$

where $v_{lb}$ and $v_{ub}$ correspond to the lower and upper limits of the estimated fluxes. Examples of FBA problems include maximizing growth rate or ATP production, minimizing uptake of nutrients, etc.

Furthermore, parsimonious FBA is then used to minimize the flux while maintaining optimum flux through the objective function (48).

### Analysis of the dynamic metabolic fluxes

We selected the most relevant metabolic pathways using a flux score, which measures the net flux throughout the individual phases. In particular, we calculated the integral of each flux multiplied by biomass and normalized with the accumulated flux of consumed hexoses as follows:


(36) $${FS}_{r}=100\;\times \;\frac{\int_{t_{A}}^{t_{B}}v_{r}(t)\cdot DW(t)}{\int_{t_{A}}^{t_{B}}\left|v_{Glx}(t)\cdot DW(t)\right|+\int_{t_{A}}^{t_{B}}\left|v_{F}(t)\cdot DW(t)\right|}$$

where ${FS}_{r}$ corresponds to the flux score for reaction r, $v_{r}(t)$ (mmol/[h · DW]) is the flux under scrutiny, $v_{Glx}(t)$ (mmol/[h · DW]) corresponds to the glucose flux, equivalently $v_{F}(t)$ (mmol/[h ⋅ DW]) corresponds to the fructose flux, and DW is the predicted dry weight biomass (g) and $t_{A}$ (h) and $t_{B}$ (h) define the interval of interest. The results correspond to the mmol of compound produced per mmol of hexoses consumed ×100 (denoted as mmol/mmolH).

Given that different species might show different phase durations, we also used normalized flux scores, denoted as ${NFS}_{r}$, corresponding to the $FS_{r}$ for each individual phase as normalized by its duration in hours.

### Numerical tools

To automatize the model calibration, we used the AMIGO2 toolbox (49). AMIGO2 is a MATLAB-based toolbox focused on dynamic model identification and optimization. AMIGO2 includes sensitivity and identifiability analyzes and offers several numerical methods for simulation and optimization. Specifically, we used CVODES (50) to solve the model and the enhanced scatter search method (eSS) (51) to optimize the parameters. AMIGO2 automatically reports the parametric confidence intervals as obtained by means of the Crammèr-Rao inequality.

To compare the discontinuous and continuous models, we performed a Kolmogorov–Smirnov test over the *R*^2^ values of both models using the ks.test included in the R package stats (52).

To solve the dFBA problem, we used the direct approach and coupled AMIGO initial value problem solver (CVODES) with COBRA Toolbox (53). We used a variable-step, variable-order Adams-Bashforth-Moulton method to solve the initial value problem defined by the system of ordinary differential equations that describe the dynamics of the extracellular metabolites. At each time step determined by this method, a pFBA problem was solved using COBRA Toolbox. It should be noted that the model can be solved using alternative dFBA software tools, such as DFBAlab Gomez et al. (25) or CD dFBA de Oliveira et al. (27), with the appropriate modification for the definition of time-dependent objective functions.

Integrals required to compute flux scores were evaluated using the standard trapezoidal method (function trapz in MATLAB).

All supplemental material and scripts necessary to reproduce the results are distributed at https://doi.org/10.5281/zenodo.14505700.

## SUPPLEMENTAL MATERIAL

Data Set S1 msystems.01615-24-s0001.xlsx Comparison between discontinuous and continuous model approaches.

Data Set S2 msystems.01615-24-s0002.xlsx Presence and absence of gene analysis between *S. cerevisiae* EC1118 and S2888c.

Supplemental material msystems.01615-24-s0003.pdf Supplemental text, figures, tables, and data set captions.

ASM does not own the copyrights to Supplemental Material that may be linked to, or accessed through, an article. The authors have granted ASM a non-exclusive, world-wide license to publish the Supplemental Material files. Please contact the corresponding author directly for reuse.
