# Supplementary material for: An integrated multiphase dynamic genome-scale model explains batch fermentations led by species of the Saccharomyces genus
Source: mSystems. 2025 Jan 22;10(2):e01615-24. doi: 10.1128/msystems.01615-24 (PMC11838008; doi:10.1128/msystems.01615-24)
Supplement: Supplemental material — Supplemental text, figures, tables, and data set captions. [file msystems.01615-24-s0003.pdf]

Supplementary Material: An integrated multiphase dynamic genome-scale model explains batch fermentations led by species of the *Saccharomyces* genus

Artai R. Moimenta , Diego Troitiño-Jordedo, et al.

Definition of the objective function in dFBA

We used a parsimonious implementation of flux balance analysis with several cellular objectives: maximizing biomass (biomass not being a constraint in this case), maximizing ATP, maximizing a combination of ATP and protein, maximizing a combination of accumulation of ATP and carbohydrates. In the following table we compare the biomass fluxes as normalized by phase duration obtained with the different static objective functions and the estimations using the kinetic model fitted to measured data:

Static objective (maximization)	Lag phase	Exponential phase	gng	Stationary phase
Biomass	3.67E-09	1.33E-08	4.03E-03	1.94E-05
ATP	0.00E+00	0.00E+00	0.00E+00	0.00E+00
0,9*ATP+0,1*prot	0.00E+00	7.94E-09	3.46E-01	1.43E-06
0,8*ATP+0,2*carb	0.00E+00	7.31E-09	1.95E-03	1.82E-06
Expected	0.00E+00	2.65E-02	2.16E-02	9.30E-05

TABLE 1 Normalized flux scores as obtained for different static objectives and dFBA as compared to the expected values obtained using the kinetic model.

As it can be concluded from the results, none of the static objective formulations succeeded. Maximizing biomass resulted in no growth during the exponential phase,

maximizing ATP resulted in lack of growth, maximizing the combination of ATP and protein underestimates growth in the exponential phase, and overestimates growth in the gng phase, while maximizing a combination of ATP and carbohydrate accumulation underestimates growth throughout the process.

Central carbon metabolism of *S. uvarum* at 25ºC as explained by the CMP model

Figure S1 presents the flux scores obtained with the CMP model for the central carbon metabolism of *S. uvarum* in batch fermentation at 25ºC. This comparison highlights how yeast cells adapt their metabolism to different environmental conditions, optimizing energy production and survival strategies.

Under anaerobic conditions, yeast primarily relies on glycolysis for ATP production. The maximum flux is observed through this pathway both during the exponential and stationary phases. However, in the exponential phase, the flux is almost twice the flux in the stationary phase. The maximum ethanol production rate is observed during the exponential phase, as expected.

The pentose phosphate pathway operates alongside glycolysis and serves two main functions: generating NADPH and producing ribose-5-phosphate for nucleotide synthesis. The figure shows how the oxidative branch is more active (around six times more active) during exponential growth. Similar differences are observed in the non-oxidative branch, although the flux through this branch is less than 25 times the flux through the oxidative path. This might be explained by considering the need to produce NADPH for redox balance.

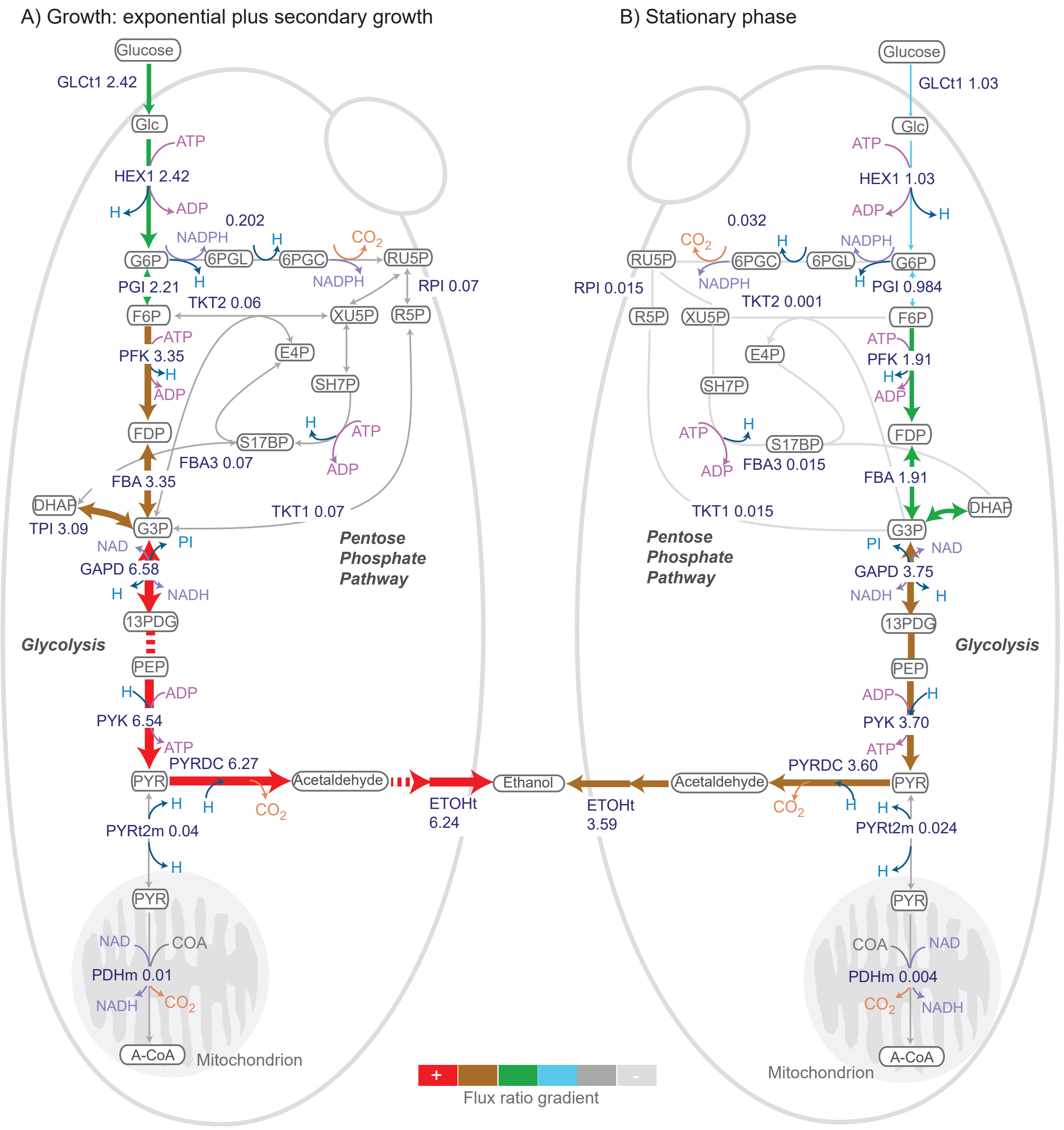

38

FIG S 1 **Central carbon metabolism of *S. uvarum* as explained by the continuous multi-phase model.** Figure shows flux scores normalized by phase duration. Figure A) presents growth, and Figure B) presents the stationary phase. Note that, as expected, metabolic activity is decreasing over time.

Model calibration, reduction and optimal parameters

In this section, we provide additional information on the calibration and reduction of the proposed model in the study.

After estimating the parameters, we found that the decay did not play a role in any of the three species, which allowed us to remove ϕ_{ED} from the model and set the cell decay rate (K_D) at 0, thus reducing the model. Furthermore, we found that inhibition of transport due to ethanol (ϕ_{Eth}) was not relevant to the quality of fit, so we removed it from the model. We also fixed the parameters associated with the biomass composition using the values reported by Schulze et al. for nitrogen-limited batch fermentation (59% protein and 12% mRNA).

The parameters associated with the uptake of amino acids (k_i) were well identified using the Nelder-Mead (simplex *fminsearch*) method implemented in the AMIGO2 toolbox. However, in those cases where amino acids have already been consumed after 21 hours, the quality of the parameter estimates, in terms of confidence intervals, was poor due to the limited amount of data in the early hours of fermentation. In this scenario, we used the same uptake constant for histidine, isoleucine, leucine, lysine, and methionine to improve identifiability. Similarly, we used the same constant for those non-preferential amino acids, e.g. cysteine and glycine. Note that the fit quality was equivalent while the confidence in the parameter estimates improved.

The optimal parameter values and the corresponding confidence intervals are reported in Tables **2** and **3**. We also include the kinetic parameters for the amino acids uptake in Table **4**.

	ScEC1118		SkCR85		SuBMV	
par	value	$\theta(\%)$	value	$\theta(\%)$	value	$\theta(\%)$
Φ_C	1.35	46.9%	$7.83 \cdot 10^{-2}$	151%	$1.27 \cdot 10^{-1}$	80.6%
$Y_{x/N}$	4.55	16.5%	8.44	15.3%	7.85	17.1%
μ_{maxN}	$2.31 \cdot 10^{-1}$	9.84%	$2.62 \cdot 10^{-1}$	13.2%	$2.77 \cdot 10^{-1}$	18.4%
Θ_C	$7.90 \cdot 10^{-1}$	4.33%	$5.51 \cdot 10^{-1}$	15.3%	$5.99 \cdot 10^{-1}$	13.6%
k_N	$7.42 \cdot 10^{-2}$	20.4%	$8.62 \cdot 10^{-2}$	33.6%	$1.26 \cdot 10^{-1}$	34.5%
$v_{max,Glx}$	1.79	17.7%	1.89	29.4%	2.84	34.3%
$v_{max,F}$	1.82	21.6%	$8.77 \cdot 10^{-1}$	18.5%	$6.17 \cdot 10^{-1}$	17.8%
ks_{Glx}	12.39	11.5%	35.91	32.7%	99.92	33.8%
ks_F	32.36	25.6%	23.79	20.4%	32.24	10.7%
α	19.26	52.2%	7.96	51.6%	11.69	28.1%
$\tau_{G_{N,S}}$	$9.99 \cdot 10^{-1}$	581%	$9.99 \cdot 10^{-1}$	$1.35 \cdot 10^{3}$%	$1.62 \cdot 10^{-2}$	724%
ks_C	$2.64 \cdot 10^{-3}$	35.7%	$1.19 \cdot 10^{-4}$	28.8%	$1.35 \cdot 10^{-3}$	21.5%
ks_S	$4.87 \cdot 10^{2}$	36.9%	63.87	45.8%	$5.00 \cdot 10^{2}$	$2.57 \cdot 10^{3}$%
a_0	$2.09 \cdot 10^{-1}$	31.8%	$1.63 \cdot 10^{-1}$	41.5%	$1.09 \cdot 10^{-1}$	63.5%
a_{02}	$4.56 \cdot 10^{-4}$	85.4%	1	—	$1.00 \cdot 10^{-4}$	172%
λ_C	0.29	—	0.29	—	0.29	—
λ_P	0.59	—	0.59	—	0.59	—
δ	1	—	1	—	1	—

TABLE 2 Biomass and nutrients uptake related parameters: optimal values and associated confidence intervals. Confidence intervals marked with the symbol — correspond to the parameters whose values were fixed during the optimization.

	ScEC1118		SkCR85		SuBMV	
par	value	$\theta(\%)$	value	$\theta(\%)$	value	$\theta(\%)$
Y_{Eth}	$4.43 \cdot 10^{-1}$	0.54%	$4.50 \cdot 10^{-1}$	2.06%	$4.48 \cdot 10^{-1}$	0.202%
Y_{Gly}	$5.89 \cdot 10^{-2}$	17.7%	$5.54 \cdot 10^{-2}$	9.41%	$5.30 \cdot 10^{-2}$	1.99%
Y_{Ace}	$7.61 \cdot 10^{-3}$	17.6%	$6.62 \cdot 10^{-3}$	9.58%	$8.09 \cdot 10^{-3}$	6.38%
Y_{Succ}	$3.77 \cdot 10^{-3}$	7.66%	$1.36 \cdot 10^{-2}$	9.79%	$3.73 \cdot 10^{-2}$	9.11%
Y_{EthylA}	$3.02 \cdot 10^{-4}$	8.5%	$3.47 \cdot 10^{-4}$	6.06%	$3.45 \cdot 10^{-4}$	19.6%
Y_{IsoA}	$7.91 \cdot 10^{-6}$	10.4%	$9.44 \cdot 10^{-6}$	8.78%	$2.20 \cdot 10^{-5}$	26.7%
$Y_{Isobutanol}$	$1.65 \cdot 10^{-4}$	23.4%	$1.42 \cdot 10^{-5}$	14.7%	$9.46 \cdot 10^{-4}$	8.18%
Y_{Iamo}	$2.34 \cdot 10^{-3}$	16.2%	$5.26 \cdot 10^{-3}$	7.63%	$5.14 \cdot 10^{-3}$	7.5%
$Y_{PhenylEthylA}$	$3.28 \cdot 10^{-5}$	29.1%	$4.21 \cdot 10^{-5}$	23.7%	$6.49 \cdot 10^{-5}$	10.4%
$Y_{PhenylEthanol}$	$7.61 \cdot 10^{-3}$	28.5%	$5.28 \cdot 10^{-3}$	24.6%	$8.29 \cdot 10^{-3}$	11.1%
$Y_{Lactate}$	$2.55 \cdot 10^{-3}$	18.4%	$2.41 \cdot 10^{-3}$	8.42%	$3.66 \cdot 10^{-3}$	5.26%
Y_{BDO}	$1.70 \cdot 10^{-2}$	28.1%	$1.28 \cdot 10^{-2}$	18.2%	$1.09 \cdot 10^{-2}$	10.5%
kc_{Mal}	$8.86 \cdot 10^{-4}$	19.2%	$1.04 \cdot 10^{-3}$	15.8%	$2.00 \cdot 10^{-4}$	27.5%
kc_{Ace}	0	—	0	—	0	—
kc_{Succ}	0	—	0	—	0	—
$k_{Sucrose}$	0	—	0	—	0	—

TABLE 3 Metabolic yield production/consumption related parameters: optimal values and associated confidence intervals. Confidence intervals marked with the symbol — correspond to the parameters whose values were fixed during the optimization.

	ScEC1118		SkCR85		SuBMV	
par	value	$\theta(\%)$	value	$\theta(\%)$	value	$\theta(\%)$
k_{Ala}	$2.61 \cdot 10^{-1}$	17.1%	$1.63 \cdot 10^{-1}$	16.6%	$1.26 \cdot 10^{-1}$	17.2%
k_{Arg}	$3.50 \cdot 10^{-1}$	17.3%	$2.14 \cdot 10^{-1}$	17.9%	$2.86 \cdot 10^{-1}$	20.8%
k_{Asp}	$6.00 \cdot 10^{-1}$	18.3%	$6.31 \cdot 10^{-1}$	22.9%	$5.22 \cdot 10^{-1}$	26.5%
k_{Glu}	$2.65 \cdot 10^{-1}$	16.7%	$2.10 \cdot 10^{-1}$	17.1%	$2.47 \cdot 10^{-1}$	18.8%
k_{Gln}	$6.10 \cdot 10^{-1}$	15.9%	$2.26 \cdot 10^{-1}$	15.8%	$3.02 \cdot 10^{-1}$	18.2%
k_{NH4Cl}	$2.91 \cdot 10^{-1}$	15.8%	$2.30 \cdot 10^{-1}$	17.3%	$1.99 \cdot 10^{-1}$	17.7%
k_{Phe}	$7.87 \cdot 10^{-1}$	19.2%	$6.17 \cdot 10^{-1}$	20%	$8.67 \cdot 10^{-1}$	67.6%
k_{Ser}	$8.50 \cdot 10^{-1}$	16.5%	$4.96 \cdot 10^{-1}$	19.7%	$5.31 \cdot 10^{-1}$	18.4%
k_{Thr}	$7.64 \cdot 10^{-1}$	16.7%	$3.94 \cdot 10^{-1}$	16.5%	$2.57 \cdot 10^{-1}$	24.9%
k_{Try}	$8.50 \cdot 10^{-1}$	16.5%	$9.00 \cdot 10^{-1}$	75.1%	$4.76 \cdot 10^{-1}$	177%
k_{Tyr}	$2.99 \cdot 10^{-1}$	22.2%	$1.27 \cdot 10^{-1}$	16.7%	$2.88 \cdot 10^{-1}$	211%
k_{Val}	$3.52 \cdot 10^{-1}$	17%	$3.82 \cdot 10^{-1}$	20.4%	$4.05 \cdot 10^{-1}$	21%
k_{AAfast}	$3.40 \cdot 10^{-1}$	39.2%	$9.45 \cdot 10^{-1}$	21.2%	$9.50 \cdot 10^{-1}$	24.5%
k_{AAlow}	$3.34 \cdot 10^{-1}$	74.5%	$3.77 \cdot 10^{-1}$	71.1%	$2.16 \cdot 10^{-1}$	161%

TABLE 4 Amino acids uptake related parameters: optimal values and associated confidence intervals. Confidence intervals marked with the symbol — correspond to the parameters whose values were fixed during the optimization.

CMP model best fit to the data

Experimental data versus the corresponding model predictions: the experimental data for all observables (dry weight, sugars, amino acids, ammonia, and primary and secondary metabolites) and sampling times are shown in the y-axis, and the corresponding predictions, in the x-axis. All pair values are in g/L. A linear regression analysis is performed (red line) and compared to the 1:1 line (blue line), which represents the perfect agreement between the predicted and experimental values to assess the accuracy of the model predictions. The model recovered all data successfully, red lines almost coincide with the blue lines and mean $R^2 \geq 0.936$ for all strains.

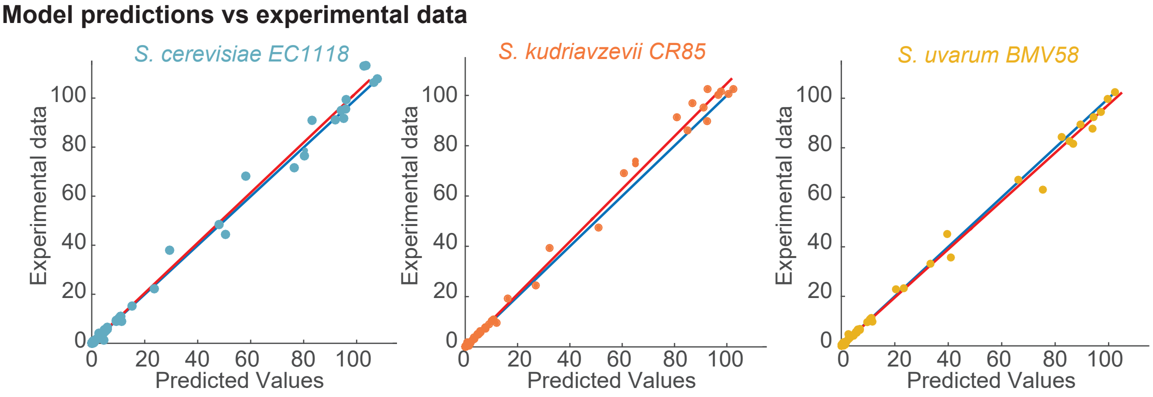

FIG S **2** Fit of the model to fermentation all time course data for batch fermentations led by three species of the *Saccharomyces* genus.

Figures S3-S5 show the best fit to the data. We determined the R-squared goodness of fit (R^2) for each measured variable and each strain-based fermentation. Most coefficients were positive with few exceptions (9 of 117), typically associated with low signal-to-noise ratio, high data dispersion, or no data observed in the measured variable. The mean R^2 (excluding negative values) was 0.96 for *S. cerevisiae EC1118*, 0.94 for *S. uvarum BMV58* and 0.94 for *S. kudriavzevii CR85*; the median R^2 values are above 0.98 for all strains.

Note that the model does not capture some specific data in the fits for SkCR85 (butanediol, isoamyl acetate, and acetate). This can be explained by a combination of two effects. In one hand, we are using the log-likelihood function in parameter estimation; therefore, we are giving more credibility to those data with lower standard deviation. In the other hand, in all these three metabolites, data would suggest certain uptake at the end of the fermentation. However, there is no sufficient evidence in the literature that this uptake is occurring, particularly in the presence of sugars. In addition, incorporating such

an uptake mechanism in the model, in the light of limited data, would render the corresponding kinetics poorly identifiable.

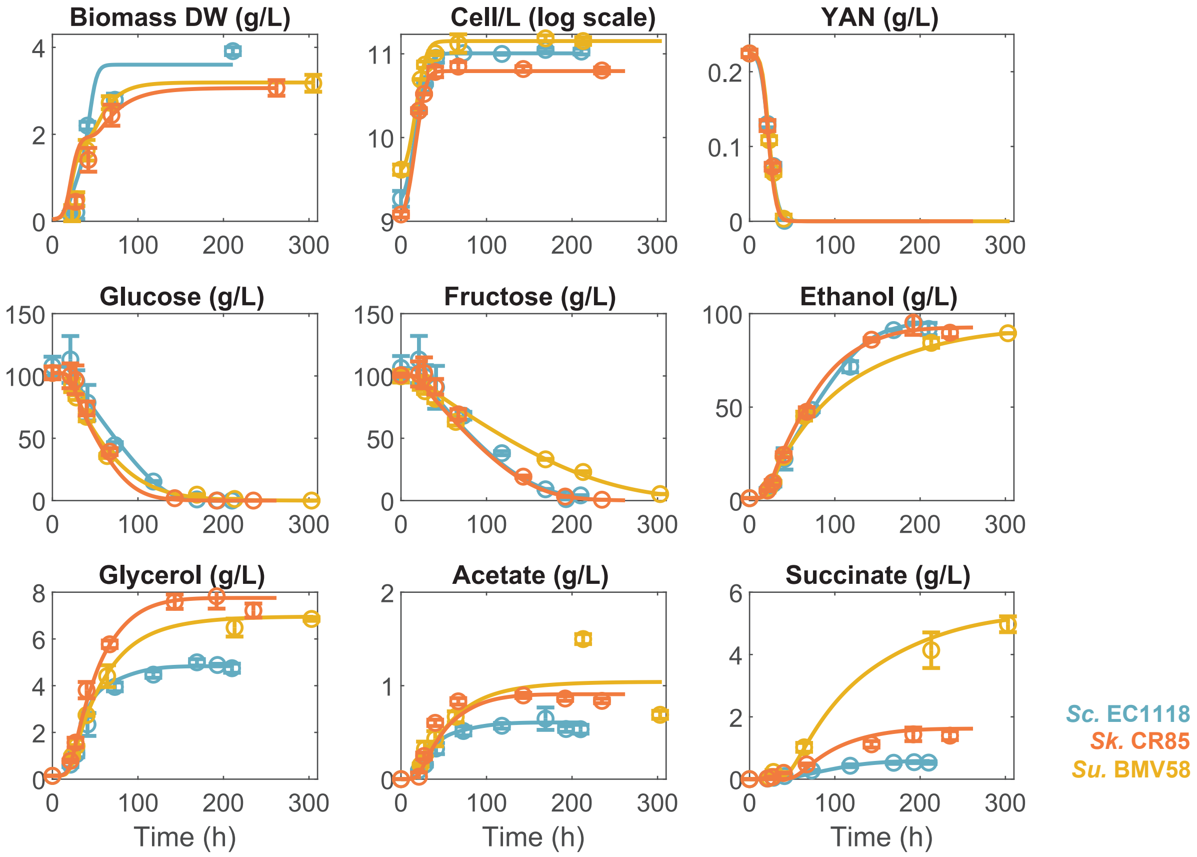

FIG S **3** Fit of the model to fermentation time course data for batch fermentations led by three species of the *Saccharomyces* genus. The figure shows the dynamics of the biomass in dry weight, viable cells in CFU, yeast assimilation nitrogen, glucose and fructose uptake and the production of ethanol, glycerol, acetate and succinate. Shading areas correspond to model uncertainty associated with the quantity and uncertainty of data.

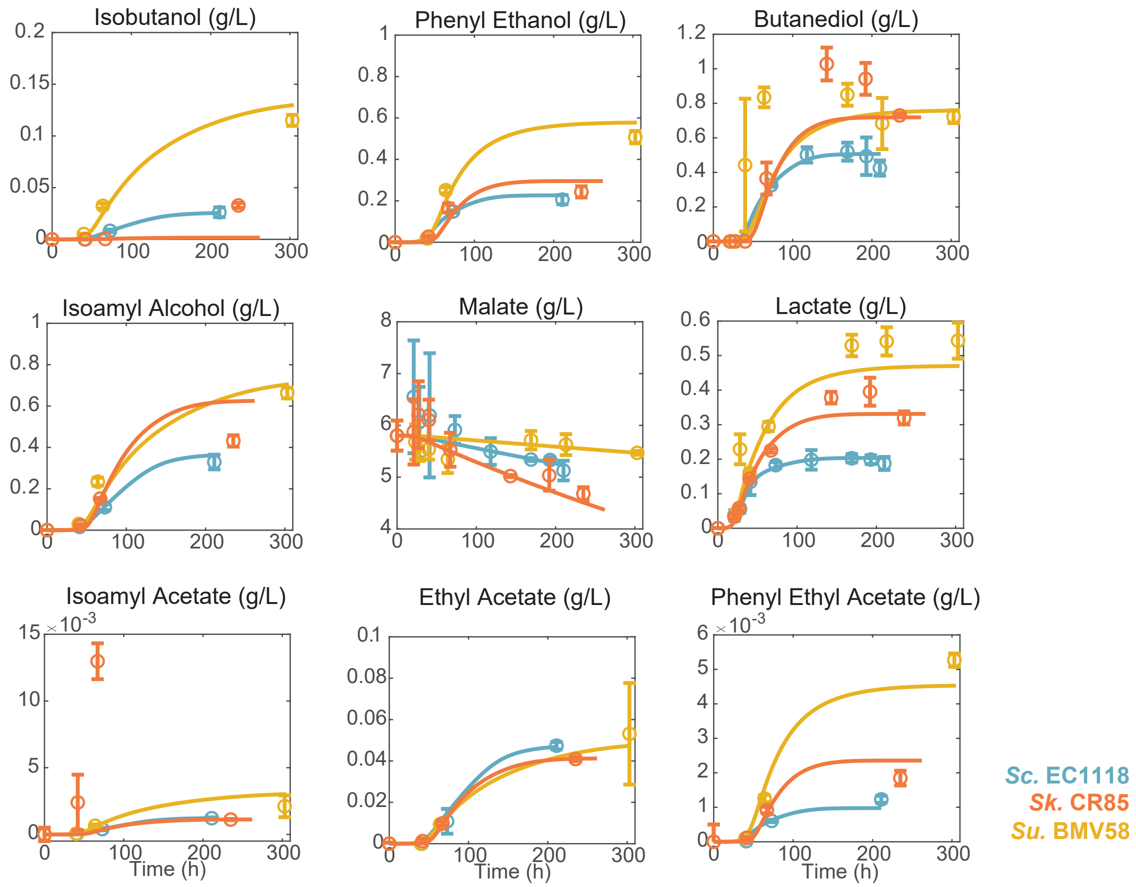

90

FIG S **4** Fit of the model to fermentation time course data for batch fermentations led by three species of the *Saccharomyces* genus. The figure shows the dynamics of the secondary metabolism products released during fermentation.

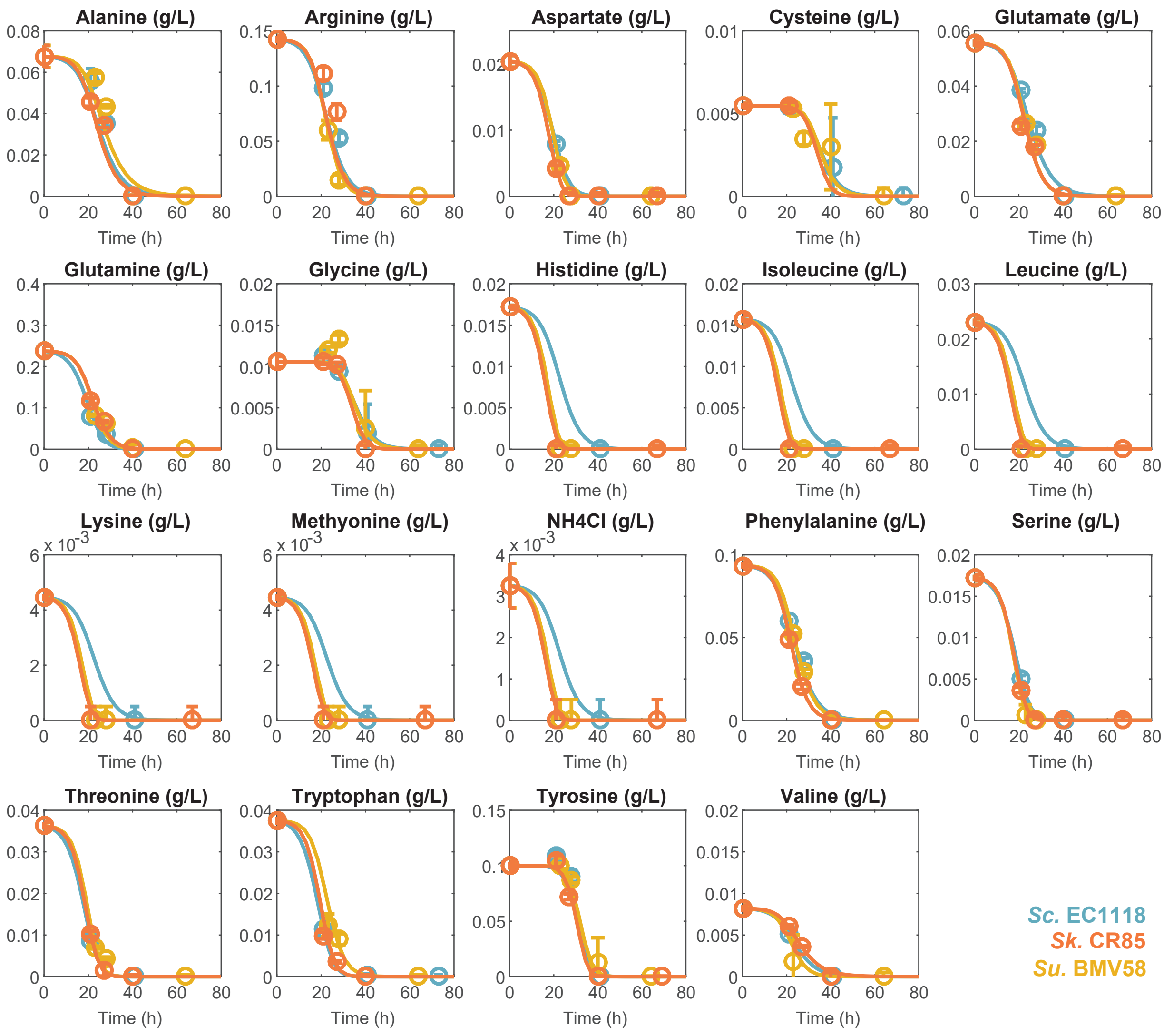

FIG S **5** Fit of the model to fermentation time course data for batch fermentations led by three species of the *Saccharomyces* genus. The figure shows the dynamics of the uptake of amino acids. Shading areas correspond to model uncertainty associated with the quantity and uncertainty of data.

Model parameters description

Par name	Par name in scripts	Units	Description
Φ_C	proportional	h^{-1}	Proportional gain parameter.
$Y_{x/N}$	YxN	g_{DW}/g_N	Nitrogen to biomass yield parameter.
μ_{MaxN}	muMaxN	h^{-1}	Maximum specific growth rate.
X_C	targetCarb	g_{DW}	Carbohydrate proportion target point parameter.
k_N	kN	g/L	Monod parameter.
k_D	Kd	h^{-1}	Cellular decay parameter.
$v_{max,Glx}$	vMaxGlx	$\frac{g/L}{h \cdot g_{DW}/L}$	Maximum rate of glucose transport parameter.
$v_{max,F}$	vMaxF	$\frac{g/L}{h \cdot g_{DW}/L}$	Maximum rate of fructose transport parameter.
ks_{Glx}	ksGlx	g/L	Michaelis-Menten constant.
ks_F	ksF	g/L	Michaelis-Menten constant.
$k_{Sucrose}$	K_sucrose	h^{-1}	Mass action parameter.
$\tau_{G_{N,S}}$	tau_gene	—	Gene regulation velocity parameter.
a_0	a0	—	Lag phase parameter.
a_{02}	a02	—	Lag phase parameter.
λ_C	optCarb	—	Percentage of carbohydrates in biomass.
λ_P	optProt	—	Percentage of proteins in biomass.
$k_{s,C}$	ks_C	g/L	YAN half-saturation parameter.
$k_{s,S}$	ks_S	g/L	Sugar half-saturation parameter.
k_{dEi}	kdE_i	g/L	Ethanol half-saturation parameter.
α	alpha	—	Percentage increase of sugar transporters.
δ	delta	—	Binary parameter to select synthetic or natural must.
kc_{Mal}	kc_Mal	h^{-1}	Mal rate consumption parameter.
kc_{Ace}	kc_Ace	h^{-1}	Acetate rate consumption parameter.
kc_{Succ}	kc_Succ	h^{-1}	Succinate rate consumption parameter.
k_{Ala}	kAla	$\frac{g/L}{h \cdot g_{DW}/L}$	Alanine mass action kinetic parameter.
k_{Arg}	kArg	$\frac{g/L}{h \cdot g_{DW}/L}$	Arginine mass action kinetic parameter.
k_{Asp}	kAsp	$\frac{g/L}{h \cdot g_{DW}/L}$	Aspartate mass action kinetic parameter.
k_{Glu}	kGlu	$\frac{g/L}{h \cdot g_{DW}/L}$	Glutamate mass action kinetic parameter.
k_{Gln}	kGln	$\frac{g/L}{h \cdot g_{DW}/L}$	Glutamine mass action kinetic parameter.
k_{NH4Cl}	kNH4Cl	$\frac{g/L}{h \cdot g_{DW}/L}$	Ammonium mass action kinetic parameter.
k_{Phe}	kPhe	$\frac{g/L}{h \cdot g_{DW}/L}$	Phenylalanine mass action kinetic parameter.
k_{Ser}	kSer	$\frac{g/L}{h \cdot g_{DW}/L}$	Serine mass action kinetic parameter.
k_{Tyr}	kTyr	$\frac{g/L}{h \cdot g_{DW}/L}$	Tyrosine mass action kinetic parameter.
k_{Try}	kTry	$\frac{g/L}{h \cdot g_{DW}/L}$	Tyriptophan mass action kinetic parameter.
k_{Thr}	kThr	$\frac{g/L}{h \cdot g_{DW}/L}$	Threonine mass action kinetic parameter.
k_{Val}	kVal	$\frac{g/L}{h \cdot g_{DW}/L}$	Valine mass action kinetic parameter.
k_{AAfast}	kAAfast	$\frac{g/L}{h \cdot g_{DW}/L}$	Fast amino acids mass action kinetic parameter.
k_{AAlow}	kAAlow	$\frac{g/L}{h \cdot g_{DW}/L}$	Slow amino acids mass action kinetic parameter.
Y_{Eth}	YEth	$gEth/g_{hexose}$	Ethanol yield.
Y_{Gly}	YGlycerol	$gGly/g_{hexose}$	Glycerol yield.
Y_{Ace}	YAce	$gAce/g_{hexose}$	Acetate yield.
Y_{Succ}	YSuccinate	$gSucc/g_{hexose}$	Succinate yield.
Y_{EthylA}	YEthylA	$gEthyAcetate/g_{hexose}$	Ethyl acetate yield.
Y_{IsoA}	YIsobutylAcetate	$gIsobutylAcetate/g_{hexose}$	Isoamyl acetate yield.
$Y_{Isobutanol}$	YIsobutanol	$gIsobutanol/g_{hexose}$	Isobutanol yield.
Y_{Iamo}	YIamo	$gIamo/g_{hexose}$	Isoamyl alcohol yield.
$Y_{PhenylEthylA}$	YPhenylEthylA	$gPhenylEthylA/g_{hexose}$	Phenyl ethyl acetate yield.
$Y_{PhenylEthanol}$	YPhenylEthanol	$gPhenylEthanol/g_{hexose}$	Phenyl ethanol yield.
$Y_{Lactate}$	YLactate	$gLactate/g_{hexose}$	Lactate yield.
Y_{BDO}	YBDO	$gBDO/g_{hexose}$	2-3 Butanedio yield.

TABLE 5 Model parameter information. *Par name* column shows the mathematical notation used in the description of the model while *Par name in scripts* column refers to the equivalent notation used in the scripts. The table also presents units and the biological meaning of all parameters.

Additional supplemental material excel files

- **Data Set S1. Comparison between discontinuous and continuous model approach. Continuous model goodness of fit.** Table S1.1 Sheet 1 presents the optimal parameter values of the continuous model for the fermentation in natural must at 25ºC with *S. uvarum BMV58* and the R-squared values as computed for each observable using both discontinuous and continuous model. Table S1.2 Sheet 2 presents the R-squared values as computed for each observable using the continuous model for the fermentation in synthetic must at 20ºC with *S. cerevisiae EC1118*, *S. uvarum BMV58* and *S. kudriavzevii CR85*. Table S1.3 Sheet 3 presents the computed AUC scores corresponding to each cellular phase to *S. uvarum BMV58* for the simulation with the continuous model in synthetic must at 25ºC.
- **Data Set S2. Presence and absence of genes analysis between *S. cerevisiae EC1118* and *S. cerevisiae S2888c*. Simulated dynamic flux scores.** Table S2.1-6 Sheet 1-6 present the overall, lag, growth and stationary dynamic flux scores respectively as mmol of produced compounds per mmol of consumed hexoses $\times$100. Table S2.7 Sheet 7 presents the flux scores over the three species at stationary phase. Fluxes above 10^{-5} are considered to be really low and were removed. Table S2.8-9 Sheet 8-9 present the dFBA simulated metabolic differences *S. cerevisiae EC1118* and *S. kudriavzevii CR85*, and *S. cerevisiae EC1118* and *S. uvarum BMV58* from Yeast8 consensus genome-scale reconstruction respectively. Table S2.10 Sheet 10 presents the normalised by ribitol intracellular metabolomic data of a set of compounds corresponding to 3 time points for the fermentation with *S. uvarum BMV58* at 25ºC with synthetic must.

References

[1] Schulze U, Lidén G, Nielsen J, Villadsen J; 1996. Physiological effects of nitrogen starvation in an anaerobic batch culture of Saccharomyces cerevisiae. Microbiology, 142(8):2299–2310.
